# Detection of bladder cancer using urinary cell-free DNA and cellular DNA

**DOI:** 10.1186/s40169-020-0257-2

**Published:** 2020-01-14

**Authors:** Zhenyu Ou, Kai Li, Ting Yang, Ying Dai, Mohan Chandra, Jun Ning, Yongli Wang, Ran Xu, Tangjie Gao, Yu Xie, Qing He, Yuanwei Li, Qin Lu, Long Wang, Zhuo Song

**Affiliations:** 1 Department of Urology, The Third Xiangya Hospital, Central South University, Changsha, 410013 Hunan China; 2 GeneTalks Biotech Co., Ltd., Changsha, 410010 Hunan China; 3 Department of Urology, Xiangya Hospital, Central South University, Changsha, 410008 Hunan China; 4 Department of Biomedical Engineering, University of Houston, Houston, TX 77204-5060 USA; 5 Department of Urology, The Second Xiangya Hospital of Central South University, Changsha, 410011 Hunan China; 6 Department of Urology, Hunan Provincial Tumor Hospital and Affiliated Tumor Hospital of Xiangya Medical School, Central South University, Changsha, 410013 Hunan China; 7 Department of Urology, Hunan Provincial People’s Hospital, Changsha, 410002 Hunan China

**Keywords:** Cell-free DNA, Next-generation sequencing, Bladder cancer, Mutation, Hematuria

## Abstract

**Background:**

The present study sought to identify a panel of DNA markers for noninvasive diagnosis using cell-free DNA (cfDNA) from urine supernatant or cellular DNA from urine sediments of hematuria patients. A panel of 48 bladder cancer-specific genes was selected. A next-generation sequencing-based assay with a cfDNA barcode-enabled single-molecule test was employed. Mutation profiles of blood, urine, and tumor sample from 16 bladder cancer patients were compared. Next, urinary cellular DNA and cfDNA were prospectively collected from 125 patients (92 bladder cancer cases and 33 controls) and analyzed using the 48-gene panel. The individual gene markers and combinations of markers were validated according to the pathology results. The mean areas under the receiver operating characteristic (ROC) curves (AUCs) obtained with the various modeling approaches were calculated and compared.

**Results:**

This pilot study of 16 bladder cancer patients demonstrated that gene mutations in urine supernatant and sediments had better concordance with cancer tissue as compared with plasma. Logistic analyses suggested two powerful combinations of genes for genetic diagnostic modeling: five genes for urine supernatant (*TERT*, *FGFR3*, *TP53*, *PIK3CA*, and *KRAS*) and seven genes for urine sediments (*TERT*, *FGFR3*, *TP53*, *HRAS*, *PIK3CA*, *KRAS*, and *ERBB2*). The accuracy of the five-gene panel and the seven-gene panel in the validation cohort yielded AUCs of 0.94 [95% confidence interval (CI) 0.91–0.97] and 0.91 (95% CI 0.86–0.96), respectively. With the addition of age and gender, the diagnostic power of the urine supernatant five-gene model and the urine sediment seven-gene model improved as the revised AUCs were 0.9656 (95% CI 0.9368–0.9944) and 0.9587 (95% CI 0.9291–0.9883).

**Conclusions:**

cfDNA from urine bears great diagnostic potential. A five-gene panel for urine supernatant and a seven-gene panel for urine sediments are promising options for identifying bladder cancer in hematuria patients.

## Background

Bladder cancer is the 9th most common malignancy and the 13th most common cause of cancer death worldwide [1, 2]. Although hematuria is a hallmark symptom of bladder cancer, only 3% to 28% of hematuria cases observed in clinical practice are due to bladder cancer [3, 4]. Cystoscopy is widely accepted as the gold standard for detecting bladder cancer. However, it is invasive, costly, and unable to achieve 100% accuracy, particularly in early-stage tumors.

Recent years, liquid biopsy has gained much attention as a non-invasive tool for cancer diagnosis and surveillance using body fluid. Due to its advantage of easy, noninvasive sampling, urine has gained the most interest over other body fluids [5]. Urine cytology and other urinary tests, such as bladder tumor antigen, nuclear matrix protein 22, or fluorescence in situ hybridization can serve as a liquid biopsy in urinary disease. Nonetheless, none of these markers have been accepted for diagnosis in routine practice due to the limited sensitivity and/or specificity at this time. Hence, the development of a reliable noninvasive bladder cancer testing method as an alternative to cystoscopy remains of great value [6].

There are a number of molecules that can be measured in urine, including cell-free DNA (cfDNA), cellular DNA, different RNA classes (e.g., microRNAs, long noncoding RNAs, messenger RNAs), proteins, and exosomes [6–13]. Previous studies have demonstrated that DNA biomarkers detected in plasma or urine could be used to predict the risk of bladder cancer in patients with hematuria [14–17]. However, the predictive accuracy is affected by genomic complexity, tumor grade, and insufficient genomic DNA. cfDNA is present extensively as degraded nucleic acid fragments in various body fluids [18–20]. The genetic alterations in urinary cfDNA are reflective of those found within tumor cells. It has been reported that malignant and benign hematuria are associated with different gene mutations [21–23]. Moreover, some studies have revealed that urinary cfDNA had a higher tumor genome burden than that of cellular DNA, which may have an influence on diagnosis efficiency. Therefore, comparing the diagnostic accuracy of potential biomarker candidates in bladder cancer would be very useful [5, 24, 25].

In this study, a set of 48 bladder cancer-related genes was assessed using next-generation sequencing (NGS) and evaluated to elucidate their potential to differentiate malignancy from benign hematuria. The mutations in the 48 genes of interest were analyzed in these four types of samples: urine supernatant, urinary sediment, plasma, and tumor tissue. The aim of this study was to compare the diagnostic accuracy of urinary cellular DNA with cfDNA for bladder cancer diagnosis in hematuria patients.

## Materials and methods

### Patients and clinical sample collection

Institutional review board approval was obtained prior to study initiation (NCT03066310) and all of the involved patients signed informed consent forms. Ninety-two patients with bladder cancer and 33 controls were enrolled. Urine, plasma, and tumor tissue samples were collected from 16 patients (14 men and two women; mean age: 67 years, range 51–84 years) with confirmed bladder cancer at Xiangya Hospital, The Second Xiangya Hospital, Hunan Provincial Tumor Hospital, and Hunan Provincial People’s Hospital between January 2017 and November 2017. Only urine samples were collected from the remaining 76 clinically diagnosed bladder cancer patients. Patients and bladder tumor characteristics are summarized in Table 1. In this study, 33 cases of nontumor bladder disease patients (20 men and 13 women; mean age: 54 years, range 21–81 years) with hematuria were included as controls. Urine was the only specimen collected from the control cases.

**Table 1:** Demographic and clinicopathologic features of the study cohorts

Demographic features	Cancer (n = 92)	Control (n = 33)	p
Average age (range), year	63 (15–89)	54 (21–81)	0.001
Gender			
Male (%)	78 (85%)	20 (61%)	
Female (%)	14 (15%)	13 (39%)	0.008

Pathology	Cancer (n = 92)	Control (n = 33)
Stage		
pTa (%)	12 (13%)	
pT1 (%)	30 (33%)	NA
pT2 (%)	28 (30%)	
pT3/T4 (%)	22 (24%)	
Grade		
Low (%)	34 (37%)	
High (%)	46 (50%)	NA
Unknown (%)	12 (13%)	

### Methods

**Fig. 1:**
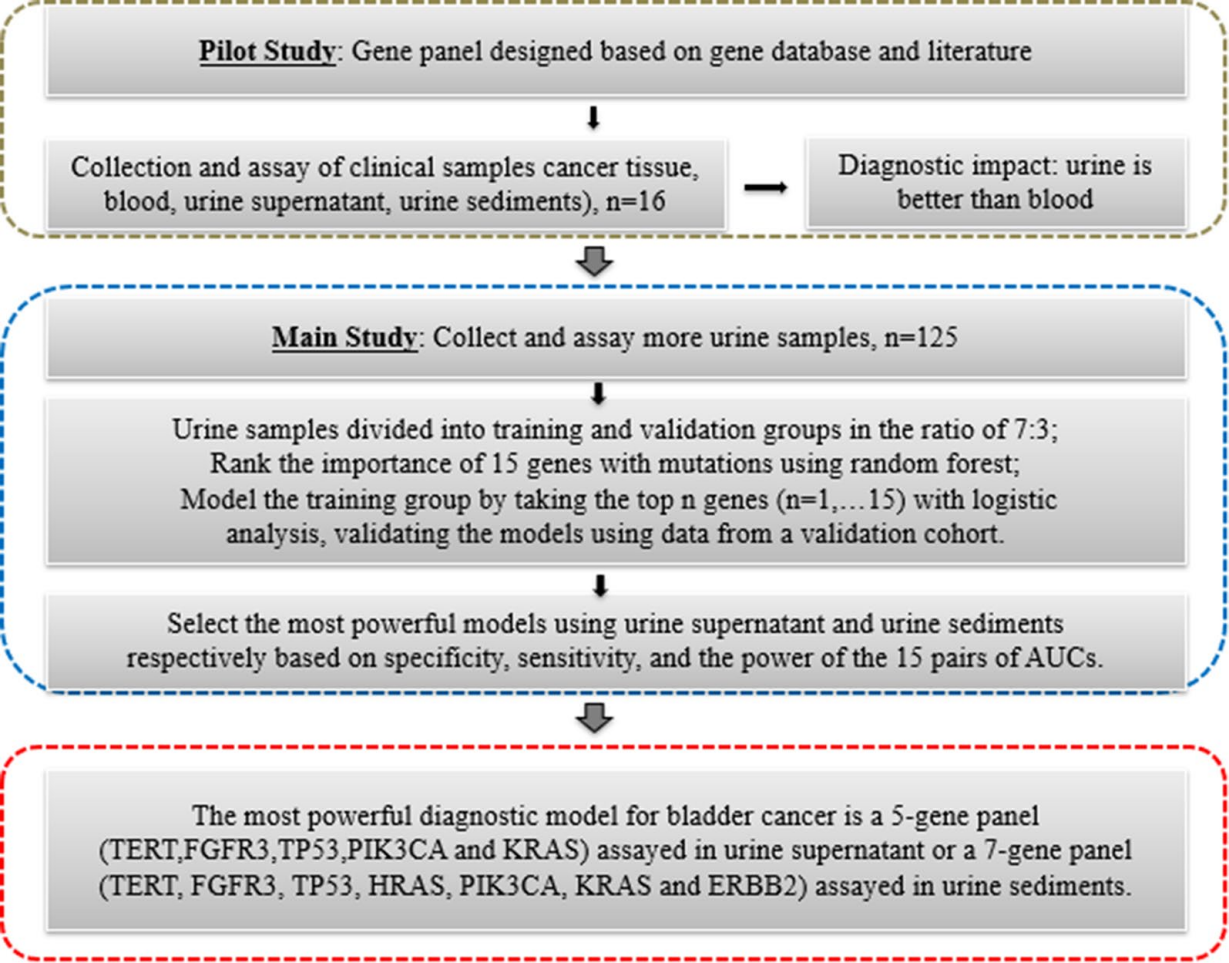
Overview of the study plan. This study consisted of three phases: a pilot study, the main study, and the finalization of the diagnostic modeling, which are shown circumscribed in grey, blue, and red, respectively

This study consisted of three phases: a pilot study, the main study, and the finalization of diagnostic modeling, which are shown circumscribed in grey, blue, and red, respectively, in Fig. 1. In the pilot study, a panel of selected genes was used to compare and select the optimal biological samples for further examination. Sixteen cases with hematuria were included in the pilot study and four biological fluids/tissues (i.e., plasma, urine supernatant, urine sediment, and cancer tissue) were tested. The main study section circumscribed in blue was designed to elucidate the concordance of mutations between the biological specimens with cancer tissues, to ascertain the gene numbers required in the gene panel optimized for the diagnostic model, and to compare the diagnostic performance of urine supernatant and urine sediments. The third and final section of the study was designed to finalize the diagnostic model.

### DNA extraction

Urine samples were collected prior to operation/cystoscopy and stored at 4 °C. Depending on the amount collected, 10–50 mL of urine was centrifuged at 1600*g* for 10 min at 4 °C. The resultant urinary supernatant and sediment were then aliquoted into new tubes. The sediment was stored at − 80 °C until assay. A total of 2 mL of urinary supernatant underwent additional high-speed centrifugation for 10 min at 12,000*g* to ensure the removal of any remaining contaminating cells and stored at − 80 °C until assay. Plasma samples were stored and processed within 72 h after collection. cfDNA from both urine and plasma were extracted using the GenMag Circulating Nucleic Acid Kit according to the manufacturer’s protocol. Tumor tissue samples were stored in a 1-mL EP tube at 80 °C for DNA isolation. Genomic DNA from urine sediment and tumor tissue was extracted using the DNeasy Blood &Tissue Kit (250; Qiagen, Hilden, Germany) according to the manufacturer’s protocol. The cfDNA and genomic DNA were quantified using Qubit3.0 and stored at − 20 °C. Genomic DNA extracted from urine sediment and tumor tissue was digested with NEBNext double-stranded DNA fragmentase (M0348) into 100- to 500-bp fragments followed by 2 × XP bead cleanup. The purified DNA was quantified using Qubit3.0.

### Selection of bladder cancer marker genes and primer design

The Cancer Genome Atlas (TCGA) (IntOGen, Goleta, CA, USA), COSMIC, My Cancer Genome, CIViC, and PubMed databases were screened for selecting bladder cancer-related genes. The 150 top-most frequent bladder cancer-related mutations from TCGA, COSMIC, as well as bladder cancer-related genes published in My Cancer Genome, CIViC, and PubMed were included in our study. In total, 48 genes were included and gene-specific primers (Additional file 1: Table S1) were designed for NGS.

### DNA mutation screening by cfBEST

For detecting low-abundance mutations in cfDNA, we developed a robust and versatile NGS-based cfDNA allelic molecule-counting system termed the cfDNA barcode-enabled single-molecule test (cfBEST). The accuracy of cfBEST was found to be comparable to that of ddPCR in a previous study [26]. Three procedures were included: prelibrary construction, sequence library (seq-library) construction, and sequencing.

During prelibrary construction, 10 ng cfDNA (or fragmented genomic DNA) was incubated with End Repair & A-Tailing Enzyme Mix and Buffer at 37 °C for 20 min and, then, 72 °C for 20 min. Adapters (Illumina, San Diego, CA, USA) harboring a barcode and flanking 30- to 40-bp sequences for further priming were ligated to the A-tailed cfDNA (or fragmented genomic DNA) at 20 °C for 15 min with the help of DNA ligase (The adapter ratio was 100:1), followed by 0.4 × XP bead cleanup. Prelibraries were amplified using a thermocycler through 10 cycles with index primers and 2 × KAPA HiFi Hot Start Ready Mix, followed by 1 × XP bead cleanup.

For seq-library construction, three consecutive amplifications with sequence-overlapped nested primers were employed as follows: PCR-1 for the enrichment of target fragments by using target primers-1 and p7, followed by 2 × XP bead cleanup and capturing with M-270 Dyna beads coated with streptavidin; PCR-2 for the repeated enrichment of target fragments, followed by 2 × XP bead cleanup; and PCR-3 using two universal primers containing P5 and P7 sequences, respectively, followed by 2 × XP bead cleanup.

For sequencing, the seq-libraries were quantified with the ABI Step One™ real-time PCR system (Thermo Fisher Scientific, Waltham, MA, USA), and then sequenced with Illumina Next-Seq 500 (Illumina, San Diego, CA, USA). Reads with 2 × 75-bp pair-end sequences were used to calculate mutation and allele ratios. The cfBEST was first calibrated using a commercial cfDNA standard template Multiplex I cfDNA Reference Standard Set, Cat. No. HD780; Horizon, Cambridge, UK) for the evaluation of sensitivity and specificity.

Next-generation sequencing data were first used to trace the unique molecules of the template to be analyzed. The unique procedures included the following steps: (1) categorization of all of the reads with the same sequences with sequencing depth; (2) identification of correct barcodes and removal of reads with wrong barcodes; (3) identification of primers and deleting reads without primer sequences; (4) blasting with the Shuman reference genome and deleting reads with any of the following features: one-sided matching, two-sided matching with mapping quality of less than 20, outside the 200 nucleotides within the target, and wrong nucleotides in primer regions; and (5) determining the unique reads in a set having four or more reads while the majority subset of the reads are at least three times more than the second-largest subset within the set sharing the same barcode. The unique molecules were further treated by trimming the two terminal nucleotides decoded with low quality by sequencing and barcode-introduced nucleotides. The remaining unique sequences were blasted with a reference sequence of the human genome (GRch37) to elucidate genetic variants using the program of BWA (version 0.7.11-r1034). The bam documents were sorted and indexed with sam tools (1.2-66-g44e1a74), then locally blasted with Genome Analysis TK (version 3.1-1-g07a4bf8). The SNP and Indel were called with samtools mpile up and annotated with annovar.

The cfBEST was used for data analysis and variant-calling. The reads with the same starting and ending positions and the same barcode reads were referred to as unique reads, and the unique reads with less than four in depth were filtered out. The average of unique reads for each sample are shown in Additional file 1: Table S2 and Additional file 2: Figure S1 (boxplot). To increase the predictive accuracy of the mutation data, inclusion criteria for a reporting mutation had to fulfill two conditions: mutation frequency of 0.005 or more and two or more unique reads [26].

### Testing and fitting the diagnostic model

Three steps were performed for the 125 pairs of urine supernatant and sediments examined. An approximate ratio of 7:3 of these samples was assigned to training and validation groups, respectively. First, the target genes with mutations identified in the samples were ordered based upon their class-predictive importance using the random forest algorithm (R version 3.2.3; R Foundation for Statistical Computing, Vienna, Austria). Second, according to the number of target genes, a logistic model was obtained using the general linear model (glm) function (R version 3.2.3): $x = a_{0} + a_{1} *gene_{1} + \cdots + a_{n} *gene_{n}$ and the model value x was then calculated. Third, the model value x was substituted into the sigmoid function $f(x) = \frac{1}{{1 + e^{ - x} }}$ to get a fit value, f(x), for diagnostic purposes. A malignancy is suggested when ${\text{f}}\left( {\text{x}} \right) > {\text{threshold}}$, while the benign state is indicated otherwise, if ${\text{f}}\left( {\text{x}} \right) \le {\text{threshold}}$.

The top n (n = 1, 2…19, respectively) of positive genes from the training group were logistically modeled, and the models derived from the training group were applied to the validation group. Each variable in the model function was repeated 100 times to ensure reproducibility. When a diagnostic model was obtained, it was then applied to the real samples in the clinical study.

### Statistics

A two-tailed t-test (R version 3.2.3) was employed for the analysis of age, number of mutations, and mutation frequencies, with a p-value of less than 0.05 considered to be statistically significant. The glm function (R version 3.2.3) was used for modeling, and the model had to show a power of more than 0.95.

## Results

### Differences in the overlap of gene mutations observed in urine supernatant, urine sediments, and plasma when compared with bladder cancer tissues

**Fig. 2:**
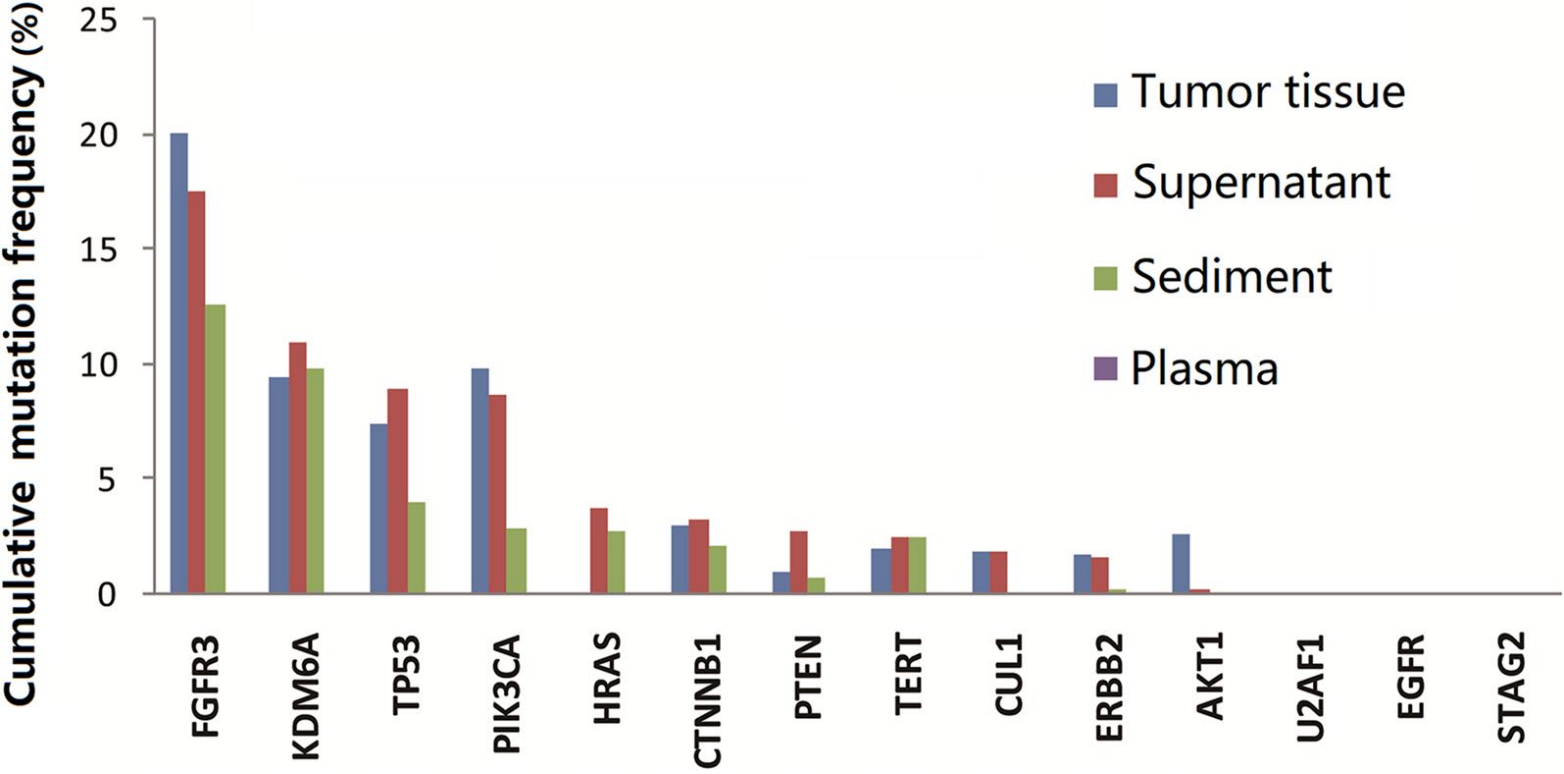
Average mutation frequencies in 14 bladder cancer biomarker genes in 16 cases in the pilot study. The cumulative frequencies of mutations identified in urine supernatant and urine sediments were comparable with those in tumor tissue and significantly higher than those in plasma

The cumulative mutation rates observed in the four types of body fluids/tissue samples from the 16 patients with hematuria were compared. As shown in Fig. 2, the cumulative mutation rate of DNA isolated from plasma was the lowest, while the cumulative mutation rates noted in urine supernatant and urine sediments were higher and closer to that of the cancer tissue. Similarly, the urine samples also showed a higher overlap of mutations relative to the cancer tissue, with the overlap being higher than that seen in the plasma samples (Additional file 3: Figure S2). The numbers of mutations identified that were identical to those seen in the cancer tissue were 24, 18, and 1, respectively, in the DNA samples isolated from urine supernatant, urine sediments, and plasma. In addition, there was no significant difference in average mutation depth among the four types of samples (Additional file 2: Figure S1). These data clearly demonstrate that urine supernatant and sediments better reflect the genetic changes in bladder cancer tissue samples as compared with the plasma and may, hence, be better suited for diagnostic purposes.

### Urine supernatant and urine sediments for genetic diagnostics of bladder cancer in an expanded cohort of hematuria samples

The above pilot study compared different body fluids/tissues, considering their diagnostic value in bladder cancer, and demonstrated the superiority of urine supernatant and sediments. We then recruited an expanded cohort of 125 cases (92 bladder cancer cases and 33 controls) with hematuria. As compared with in the pilot study of 16 cases in which positive mutations (i.e., shared with the paired cancer tissue) were identified in 14 and 10 genes, the number of genes with mutations identified in the 125 cases were 19 and 15 genes, respectively, in urine supernatant and urine sediments (Additional file 4: Figure S3). The kappa values of the detection rates of supernatant and sediment samples with gene variations are shown in Table 2. Only 9 of 92 cancer samples had a kappa value of 0, while 10 of the 33 control samples showed the same, which indicated that the consistency of supernatant and sediment samples in the cancer group achieved better performance than in the control group. The kappa values of the detection rates of different genes are shown in Table 3, where the *ACTB*, *CUL1*, *EGFR*, and *U2AF1* genes were only detected in a portion of urine supernatant samples.

**Table 2:** The kappa values of supernatant and sediment samples

Group	Kappa = 1	NaN	Kappa = 0	Kappa (0,1)	All
All samples	49	26	19	31	125
Cancer	48	4	9	31	92
Control	1	22	10	0	33

*NaN* no mutation was detected in the supernatant or sediment

**Table 3:** The kappa values of each gene among the 125 supernatant and sediment samples

Gene	Supernatant sensitivity	Supernatant specificity	Sediment sensitivity	Sediment specificity	p-value	Kappa
*TERT*	0.46	1.00	0.48	1.00	0.90	0.86
*FGFR3*	0.38	0.97	0.37	0.97	1.00	0.82
*TP53*	0.29	1.00	0.22	0.82	0.13	0.69
*PIK3CA*	0.26	0.94	0.24	0.97	1.00	0.67
*KRAS*	0.16	1.00	0.15	1.00	1.00	0.96
*CDKN2A*	0.10	1.00	0.10	1.00	1.00	1.00
*HRAS*	0.10	0.97	0.14	1.00	0.60	0.76
*AKT1*	0.08	1.00	0.08	1.00	1.00	1.00
*ERBB2*	0.07	1.00	0.07	1.00	1.00	1.00
*ACTB*	0.04	1.00	0.00	1.00	0.67	0.00
*BRAF*	0.03	1.00	0.03	1.00	1.00	1.00
*KDM6A*	0.03	0.97	0.02	1.00	1.00	0.66
*EGFR*	0.02	1.00	0.00	1.00	0.89	0.00
*CTNNB1*	0.01	1.00	0.01	1.00	1.00	1.00
*FBXW7*	0.01	1.00	0.01	1.00	1.00	1.00
*PTEN*	0.01	1.00	0.01	1.00	1.00	1.00
*STAG2*	0.01	1.00	0.01	1.00	1.00	− 0.01
*CUL1*	0.01	1.00	0.00	1.00	1.00	0.00
*U2AF1*	0.01	0.97	0.00	1.00	1.00	0.00

**Fig. 3:**
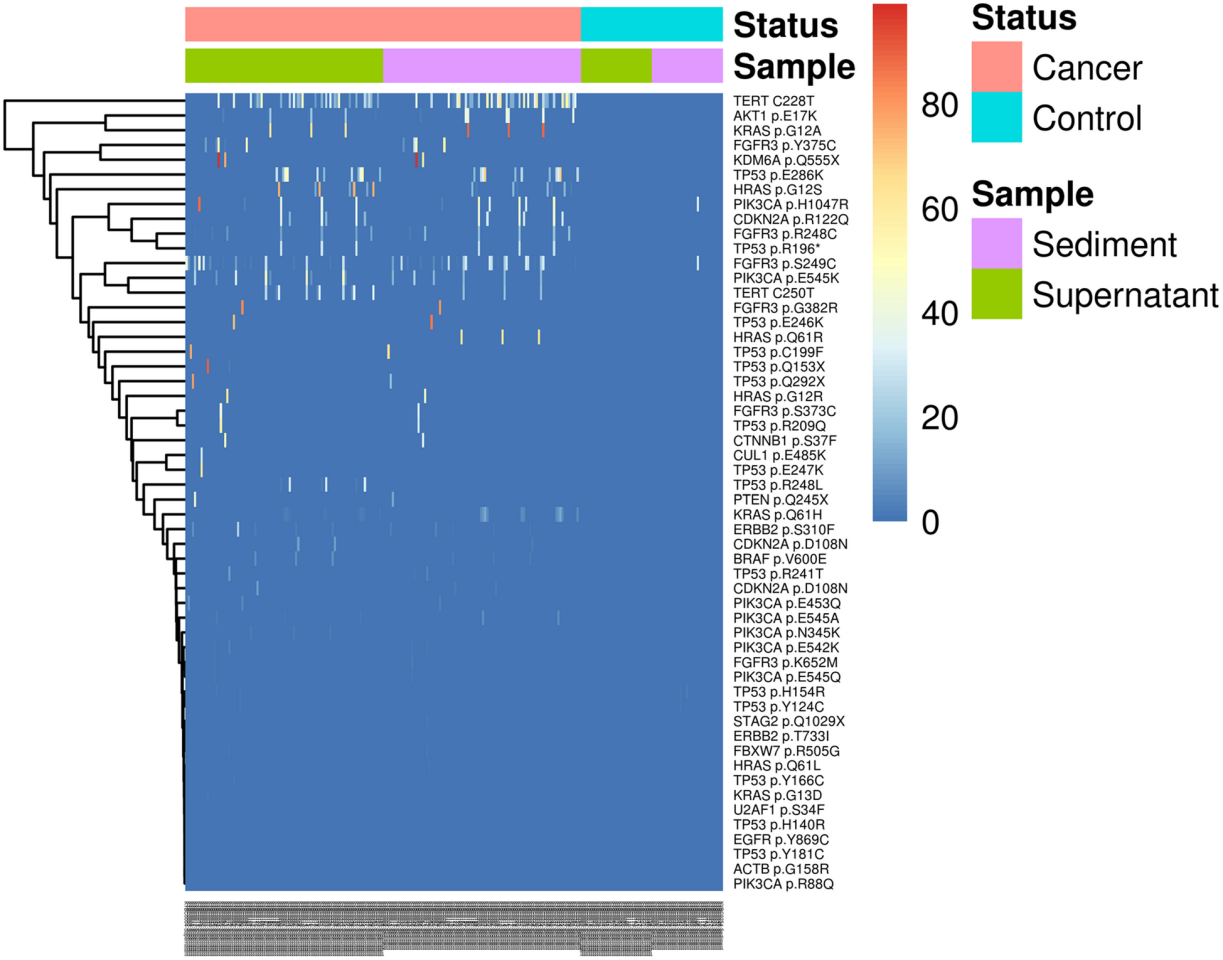
Heatmap illustrating the distribution of mutation rates identified in the DNA of urine supernatant and urine sediments. In cancer tissue, high mutation rates were identified in *KDM6A* p.Q555X, *KRAS* pG2A, *TP53* p.Q153X, *TERT* promoter, *FGFR3* p.Y375C, and *TP53* p.E246K. The Y-axis lists the mutation locations and the X-axis shows the identification number of each sample. The mutation rates from low to high are represented by their colors from blue (low) to red (high) accordingly

**Fig. 4:**
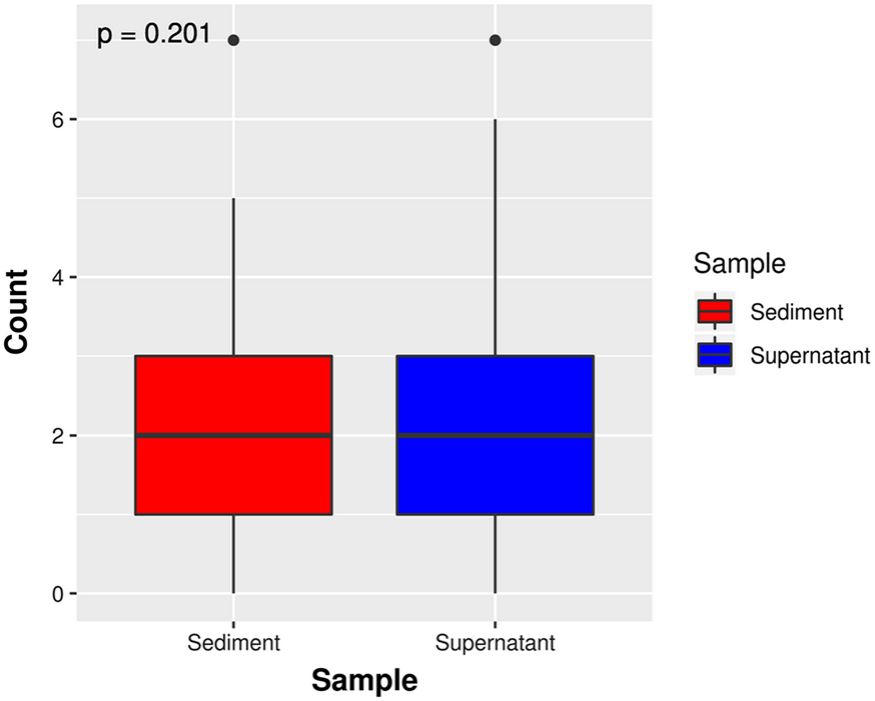
Boxplot illustrating the frequency of mutations in the urine supernatant and sediments from 92 cases. The boxplot shows the number of positive mutant genes among 92 tumor patients, whose urine supernatant and sediment samples were screened using the 48-gene panel. The points in the graph represent discrete points that were statistically distant from the median. The Y-axis represents the number of mutated genes in the sample. A t-test analysis revealed that there was no statistically significant difference in the number of mutated genes between the urine supernatant and sediments obtained from the same subjects (p = 0.201)

For the 15 genes with mutations shared by both the urine supernatant and sediments, genes with relatively higher mutation rates in cancer patients nearly overlapped in both samples, including genes, such as *TERT*, *FGFR3*, *TP53*, *PIK3CA*, and KRAS (Table 4). As is illustrated in Fig. 3, there was a high mutation relevance ratio for urine supernatant and urine sediments of the cancer samples, while few mutations were identified in the controls. However, mutations PIK3CA p.H1047R and FGFR3 p.S249C were found at a high frequency among urine sediments in the normal sample. As plotted in Fig. 4, there were no significant differences in mutations in the urine supernatant relative to in the paired urine sediments (p = 0.201 by t-test).

**Table 4:** Gene mutation detection in urine supernatant and urine sediments

Supernatant	Cancer (n = 92)		Control (n = 33)		PPV (%)	NPV (%)
	n	Sensitivity (%)	n	Specificity (%)		
*TERT*	42	46	0	100	100	40
*FGFR3*	35	38	0	100	100	37
*TP53*	27	29	0	100	100	34
*PIK3CA*	24	26	0	100	100	33
*KRAS*	15	16	0	100	100	30
*CDKN2A*	9	10	0	100	100	28
*HRAS*	9	10	0	100	100	28
*AKT1*	7	8	0	100	100	28
*ERBB2*	6	7	0	100	100	28
*ACTB*	4	4	1	97	80	27
*BRAF*	3	3	1	97	75	26
*KDM6A*	3	3	1	97	75	26
*EGFR*	2	2	0	100	100	27
*CTNNB1*	1	1	2	94	33	25
*CUL1*	1	1	0	100	100	27
*FBXW7*	1	1	0	100	100	27
*PTEN*	1	1	0	100	100	27
*STAG2*	1	1	0	100	100	27
*U2AF1*	1	1	1	97	50	26

Sediment	Cancer (n = 92)		Control (n = 33)		PPV (%)	NPV (%)
	n	Sensitivity (%)	n	Specificity (%)		
*TERT*	44	48	0	100	100	41
*FGFR3*	34	37	1	97	97	36
*PIK3CA*	22	24	1	97	96	31
*TP53*	20	22	6	82	77	27
*KRAS*	14	15	0	100	100	30
*HRAS*	13	14	0	100	100	29
*CDKN2A*	9	10	0	100	100	28
*AKT1*	7	8	0	100	100	28
*ERBB2*	6	7	0	100	100	28
*BRAF*	3	3	0	100	100	27
*KDM6A*	2	2	0	100	100	27
*CTNNB1*	1	1	0	100	100	27
*FBXW7*	1	1	0	100	100	27
*PTEN*	1	1	0	100	100	27
*STAG2*	1	1	0	100	100	27

n: The number of samples in which the gene mutation was detected

### Diagnostic model based on urine supernatant and urine sediments

**Fig. 5:**
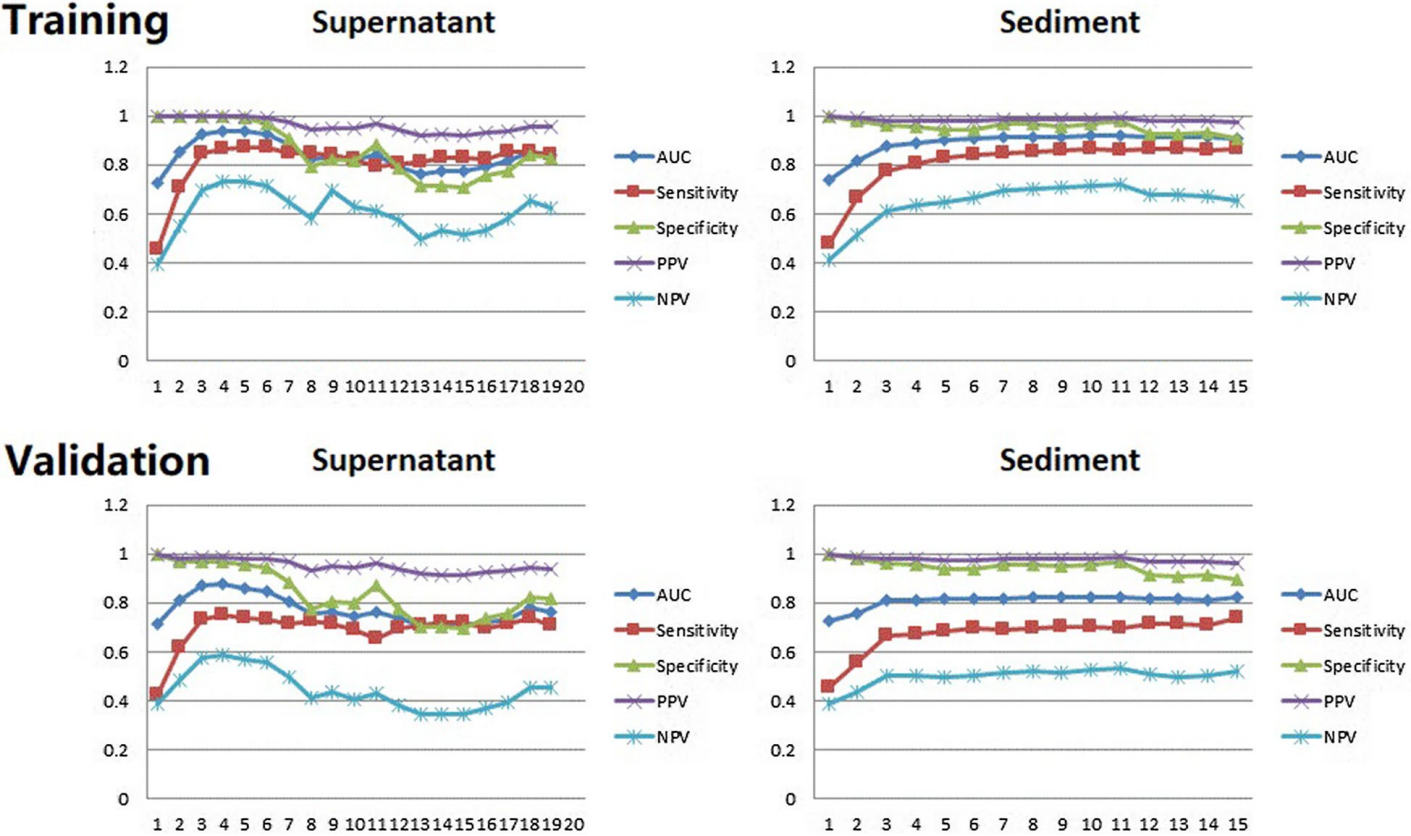
Averaged diagnostic parameters of the logistic models for the genetic diagnosis of bladder cancer. For urine supernatant, all parameters of the models are satisfactory when gene combinations having three, four, and five genes (*TERT*, *FGFR3*, *TP53*, *PIK3CA,* and *KRAS*) were used. In contrast, the models using urine sediments require the combination of seven (*TERT*, *FGFR3*, *TP53*, *HRAS*, *PIK3CA*, *KRAS,* and *ERBB2*) or more genes to attain satisfactory predictive potential. The X-axis represents the number of genes factored into the model. The Y-axis plots diagnostic potential, with 1.0 representing perfect disease discrimination

The genes with positive mutations (i.e., shared with the paired cancer tissue) were ranked by employing random forest analysis using the mutation data from urine supernatant and sediments. Logistic models were developed using the training group and then tested in the validation group. The detailed parameters used in these analyses are listed in Additional file 1: Table S3. These logistic analyses highlighted two powerful combinations of genes for genetic diagnostic modeling: five genes for urine supernatant (*TERT*, *FGFR3*, *TP53*, *PIK3CA,* and *KRAS*) and seven genes for urine sediments (*TERT*, *FGFR3*, *TP53*, *HRAS*, *PIK3CA*, *KRAS*, and *ERBB2*). As shown in Fig. 5, all four diagnostic parameters in areas under the receiver operating characteristic (ROC) curves (AUCs) using urine supernatant nearly reached their plateaus when the combination included five genes, while they definitively reached the plateaus when the combination included seven genes for the urine sediments.

**Fig. 6:**
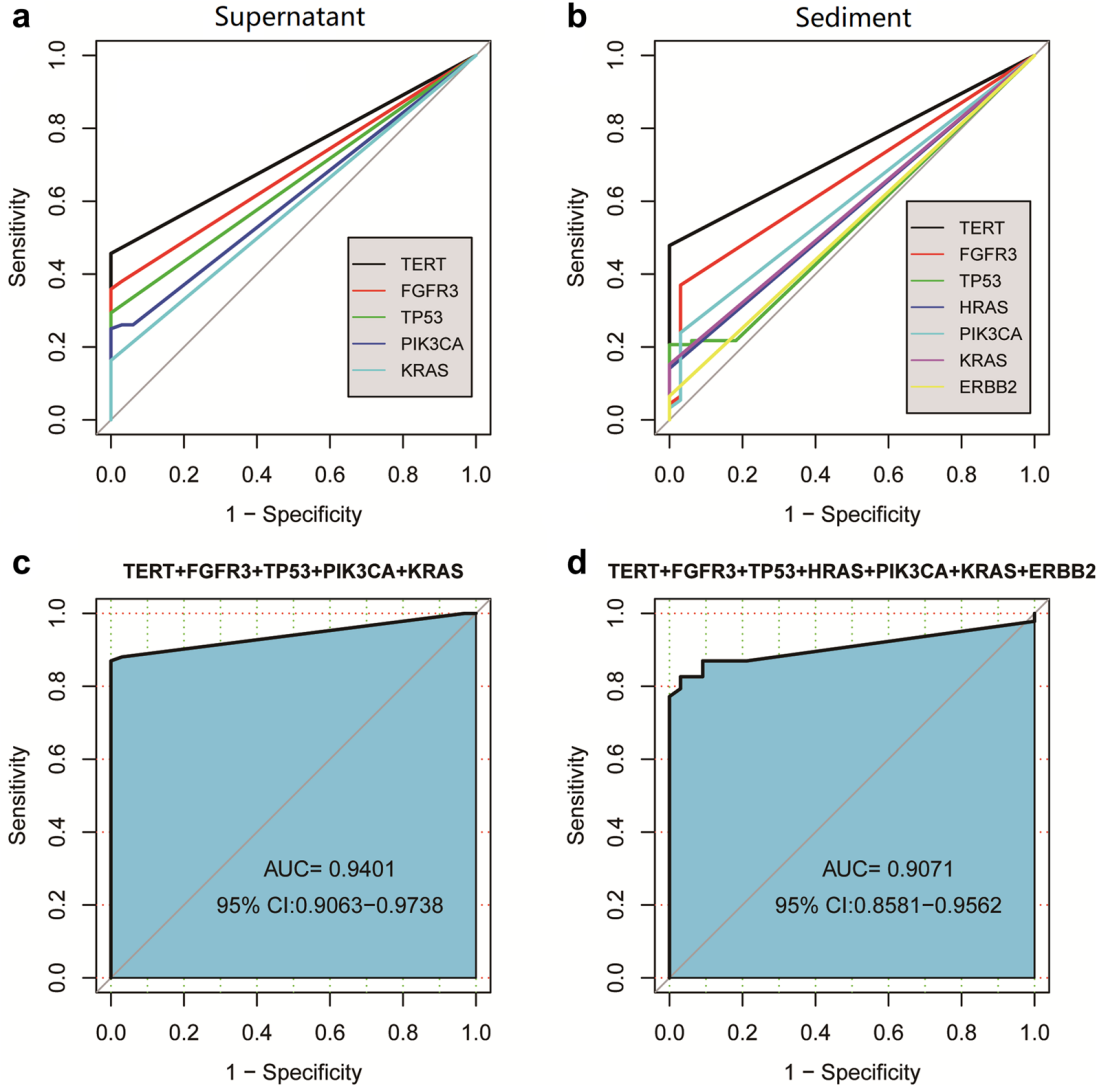
Diagnostic models based on mutations of a single gene or gene combination found in all 125 urine supernatant or urine sediment samples for discriminating cancer from controls. **a** Individual AUCs based on the five genes *TERT*, *FGFR3*, *TP53*, *PIK3CA*, and *KRAS* assayed in urine supernatant; **b** individual AUCs based on the seven genes *TERT*, *FGFR3*, *TP53*, *HRAS*, *PIK3CA*, *KRAS,* and ERBB2 assayed in urine sediments; **c**, **d** AUCs of the five-gene and seven-gene combinations using urine supernatant and sediments, respectively

After identifying the two combinations of genes useful in genetic diagnostic modeling, a serial calculation of AUCs for individual and different combinations of genes was performed (Additional file 1: Table S4 and Fig. 6). Among the AUCs derived using the 125 urine samples, the AUC of a five-gene panel from urine supernatant [AUC: 0.94; (95% confidence interval (CI) 0.91–0.97] (Fig. 6c) and that of a seven-gene panel from urine sediments (AUC: 0.91, 95% CI 0.86–0.96) (Fig. 6d) performed better than all of the others.

## Discussion

This study compares the diagnostic potential of urine supernatant, urine sediment, and plasma with tumor tissue samples obtained from the same subjects in the identification of malignancy in patients presenting with hematuria. In total, 48 bladder cancer-related candidate genes were analyzed in these four types of specimens. The cfDNA mutations identified in the urine supernatant and sediment were found to be the richest in comparison with plasma samples drawn from the same cases analyzed. Bioinformatics analysis of the urinary DNA mutation information yield diagnostic models consisting of five target genes (TERT, FGFR3, TP53, PIK3CA, and KRAS) and seven genes (TERT, FGFR3, TP53, HRAS, PIK3CA, KRAS, PIK3CA, and KRAS), using urine supernatant or urine sediments respectively, for the successful identification of malignancy in patients with hematuria.

The number and frequency of mutations among different biological samples, such as plasma, urine supernatant, and urine sediment, can be variable depending on the type and presence of metastasis of bladder cancer. Although the cases were limited, the data from the study demonstrated that the urine of patients with malignant bladder cancer showed the highest total number of mutations, with the normal urine sample exhibiting the lowest average number of total mutations. Although the total number of mutations is informative, it could not be directly used for diagnostics as these totals overlapped between patients suffering from malignant bladder cancers and the controls.

In the pilot study of 16 cases, *FGFR3* ranked the highest in terms of cumulative mutation frequency and this was subsequently validated in the expanded cohort of 125 cases; hence, this gene was included in both the five-gene and seven-gene diagnostic panels. In the pilot analysis comparing mutations from different specimens, the second richest source of mutations was observed in the gene *KDM6A* [22]. However, when the cohort was expanded to 125 cases, cumulative mutations in *KDM6A* were no longer elevated in bladder cancer samples so this gene was not used for construction of the diagnostic panel.

As a driving gene in bladder cancer, *TERT* mutations have been suggested to be useful in the genetic diagnosis and monitoring of bladder cancer recurrence [22, 27, 28]. Overall, *TERT* mutations can be found in about 50% of bladder cancers (COSMIC database) and the mutation rate could be as high as 70% [28]. In addition to its involvement in the development of bladder cancer, mutations in the *TERT* reporter region have been used in the screening of other cancer types, such as lung cancer [29]. As illustrated in Fig. 3, high rates of the *TERT* promoter-region mutation C228T were observed in the tested samples.

The present study narrowed the 48 candidate genes down to five genes or seven genes in our diagnostic models for identifying malignancy in subjects with hematuria. Considering the 12 false negatives (Additional file 1: Table S5) identified in this analysis, more biomarkers are still required for further screening as the 12 false negatives could still not be correctly diagnosed even when using the entire panel of 48 genes. Another possible explanation for the false negatives might be variations in genes involved in carcinogenesis in different subpopulations of patients from whom the data were obtained. Furthermore, the integration of more cancer-related biomarkers may increase the sensitivities and specificities in the genetic diagnosis of bladder cancer. The combination of mutation analysis and methylation assays could substantially increase the power in the genetic diagnosis of bladder cancer as well [30]. In a study with 31 cases, 24 reported an AUC of 0.96 (95% CI 0.92–0.99) with a sensitivity of 93% and specificity of 86% when the mutation analysis covered *FGFR3*, *TERT,* and *HRAS* as well as when the methylation assay covered the *OTX1*, *ONECUT2,* and *TWIST1* genes [14]. Additionally, RNAs in the urine may also serve as cancer biomarkers [9, 11, 31]. These epigenetic and RNA biomarkers should be evaluated in future research with larger sample sizes.

Clinical information, including subject age and gender, was integrated into the diagnostic models developed using the urine supernatant and sediments. We have tested the possible impact of adding age and gender in improving the diagnostic power of our model based on the genetic information. As shown in Additional file 5: Figure S4, the AUC of 0.94 (95% CI 0.91–0.97) associated with the five-gene model using urine supernatant improved slightly to 0.97 (95% CI 0.94–0.99) when demographic information was added. With the addition of age and gender, the diagnostic power of the urine sediment seven-gene model improved, showing an AUC of 0.96 (95% CI 0.93–0.99). No significant differences were found between staging, gender, and age when we tested the model performance with different staging data, different genders, and different ages (Additional file 1: Tables S6–S8). Additionally, the five-gene model of urine supernatant showed no significant differences in comparison with the urine sediment seven-gene model (Additional file 1: Table S9).

## Conclusions

In conclusion, the diagnostic value of urine genetic analysis in precision medicine of bladder cancer has great clinical potential. The high sensitivity of the diagnostic model obtained from the bioinformatic analysis may help reduce or avoid the use of cystoscopy examination for those who present with hematuria. Regardless of the false negatives, the high sensitivity and specificity of the urine five-gene and seven-gene models for identifying malignant hematuria may also have great potential for surveillance screening for bladder cancer in patients without hematuria in addition to practical application in hematuria patients.

## Supplementary information

**Additional file 1: Table S1.** Primers list. **Table S2.** Average of unique reads for each sample. **Table S3.** The diagnostic parameters for the diagnostic model with increasing numbers of genes used. **Table S4.** AUC values for each gene, in discriminating cancer from controls. **Table S5.** Basic information of 12 false-negative samples. **Table S6.** The results of a consistent comparison in different cancer stage. **Table S7.** The results of a consistent comparison in different gender. **Table S8.** The results of a consistent comparison in different age. **Table S9.** The results of a consistent comparison for 5-gene model (Supernatant) and 7 gene model (Sediment).

**Additional file 2: Figure S1.** The average mutation depth of four types of samples.

**Additional file 3: Figure S2.** The mutations identified in urine supernatant, urine sediments, plasma, and cancer tissue.

**Additional file 4: Figure S3.** Cumulative mutation rates of DNA isolated from urine supernatant and sediments in 125 cases with hematuria. These mutations were noted in 19 and 15 genes, respectively, in urine supernatant and sediments.

**Additional file 5: Figure S4.** The AUCs of urine supernatant five-gene and urine sediment seven-gene panels, respectively, in combination with integrated demographic information. With the addition of age and gender, the diagnostic power of the urine supernatant five-gene model slightly improved as the AUC reached 0.9656 (95% CI 0.9368–0.9944) and the diagnostic power of the urine sediment seven-gene model improved as the AUC reached 0.9587 (95% CI 0.9291–0.9883).

## Data Availability

The datasets used and/or analysed during the current study are available from the corresponding author on reasonable request.

## Abbreviations

cfDNA: cell-free DNA
cfBEST: cell-free DNA barcode-enabled single-molecule test
NGS: next-generation sequencing
ROC: receiver operating characteristic
AUC: area under the curve of ROC
FGFR3: fibroblast growth factor receptor 3
TP53: tumor protein P53
PIK3CA: phosphatidylinositol-4,5-bisphosphate 3-kinase catalytic subunit alpha
ERBB2: Erb-B2 receptor tyrosine kinase 2

## References

[1] Jemal A (2006). Cancer statistics, 2006. CA Cancer J Clin.

[2] Ferlay J (2015). Cancer incidence and mortality worldwide: sources, methods and major patterns in GLOBOCAN 2012. Int J Cancer.

[3] Price SJ, Shephard EA, Stapley SA, Barraclough K, Hamilton WT (2014). Non-visible versus visible haematuria and bladder cancer risk: a study of electronic records in primary care. Br J Gen Pract.

[4] Khadra MH, Pickard RS, Charlton M, Powell PH, Neal DE (2000). A prospective analysis of 1,930 patients with hematuria to evaluate current diagnostic practice. J Urol.

[5] Ward DG, Bryan RT (2017). Liquid biopsies for bladder cancer. Transl Androl Urol.

[6] Di Meo A, Bartlett J, Cheng Y, Pasic MD, Yousef GM (2017). Liquid biopsy: a step forward towards precision medicine in urologic malignancies. Mol Cancer.

[7] Abbosh PH, Rosenberg JE, Plimack ER (2016). Circulating biomarkers to guide systemic therapy for urothelial carcinoma. Urol Oncol.

[8] Cohen JD, Li L, Wang Y, Thoburn C, Afsari B, Danilova L, Douville C, Javed AA (2018). Detection and localization of surgically resectable cancers with a multi-analyte blood test. Science.

[9] Pardini B, Cordero F, Naccarati A (2018). microRNA profiles in urine by next-generation sequencing can stratify bladder cancer subtypes. Oncotarget.

[10] Birkenkamp-Demtroder K, Nordentoft I, Christensen E (2016). Genomic alterations in liquid biopsies from patients with bladder cancer. Eur Urol.

[11] Juracek J (2018). Genome-wide identification of urinary cell-free microRNAs for non-invasive detection of bladder cancer. J Cell Mol Med.

[12] Leiblich A (2017). Recent developments in the search for urinary biomarkers in bladder cancer. Curr Urol Rep.

[13] Frantzi M, Van Kessel K, Zwarthoff EC (2016). Development and validation of urine-based peptide biomarker panels for detecting bladder cancer in a multi-center study. Clin Cancer Res.

[14] van Kessel KE (2017). Validation of a DNA methylation-mutation urine assay to select patients with hematuria for cystoscopy. J Urol.

[15] Dahmcke CM (2016). A prospective blinded evaluation of urine-DNA testing for detection of urothelial bladder carcinoma in patients with gross hematuria. Eur Urol.

[16] Khetrapal P (2018). The role of circulating tumour cells and nucleic acids in blood for the detection of bladder cancer: a systematic review. Cancer Treat Rev.

[17] Lin SY, Linehan JA, Wilson TG, Hoon DSB (2017). Emerging utility of urinary cell-free nucleic acid biomarkers for prostate, bladder, and renal cancers. Eur Urol Focus.

[18] Casadio V (2013). Urine cell-free DNA integrity as a marker for early bladder cancer diagnosis: preliminary data. Urol Oncol.

[19] Zancan M (2009). Evaluation of cell-free DNA in urine as a marker for bladder cancer diagnosis. Int J Biol Markers.

[20] Zhong XY (2001). Cell-free DNA in urine: a marker for kidney graft rejection, but not for prenatal diagnosis?. Ann N Y Acad Sci.

[21] Tuononen K (2013). Comparison of targeted next-generation sequencing (NGS) and real-time PCR in the detection of EGFR, KRAS, and BRAF mutations on formalin-fixed, paraffin-embedded tumor material of non-small cell lung carcinoma-superiority of NGS. Genes Chromosom Cancer.

[22] Nickerson ML (2014). Concurrent alterations in TERT, KDM6A, and the BRCA pathway in bladder cancer. Clin Cancer Res.

[23] Christensen E (2018). Optimized targeted sequencing of cell-free plasma DNA from bladder cancer patients. Sci Rep.

[24] Robertson AG (2017). Comprehensive molecular characterization of muscle-invasive bladder cancer. Cell.

[25] Wu S (2014). Telomerase reverse transcriptase gene promoter mutations help discern the origin of urogenital tumors: a genomic and molecular study. Eur Urol.

[26] Yang X, Zhou Q, Zhou W (2019). A cell-free DNA barcode-enabled single-molecule test for noninvasive prenatal diagnosis of monogenic disorders: application to β-thalassemia. Adv Sci.

[27] Cowan M, Springer S, Nguyen D (2016). High prevalence of TERT promoter mutations in primary squamous cell carcinoma of the urinary bladder. Mod Pathol.

[28] Russo IJ (2018). Toward personalised liquid biopsies for urothelial carcinoma: characterisation of ddPCR and urinary cfDNA for the detection of the TERT 228 G > A/T mutation. Bladder Cancer.

[29] Hulbert A, Jusue-Torres I, Stark A (2017). Early detection of lung cancer using DNA promoter hypermethylation in plasma and sputum. Clin Cancer Res.

[30] Gormally E, Caboux E, Vineis P, Hainaut P (2007). Circulating free DNA in plasma or serum as biomarker of carcinogenesis: practical aspects and biological significance. Mutat Res.

[31] Sin MLY, Mach K, Sinha R (2017). Deep sequencing of urinary RNAs for bladder cancer molecular diagnostics. Clin Cancer Res.

