# Supplementary material for: Detection of bladder cancer using urinary cell-free DNA and cellular DNA
Source: Clin Transl Med. 2020 Jan 14;9:4. doi: 10.1186/s40169-020-0257-2 (PMC6960275; doi:10.1186/s40169-020-0257-2)
Supplement: Supplementary file 1 — Additional file 1: Table S1. Primers list. Table S2. Average of unique reads for each sample. Table S3. The diagnostic parameters for the diagnostic model with increasing numbers of genes used. Table S4. AUC values for each gene, in discriminating cancer from controls. Table S5. Basic information of 12 false-negative samples. Table S6. The results of a consistent comparison in different cancer stage. Table S7. The results of a consistent comparison in different gender. Table S8. The results of a consistent comparison in different age. Table S9. The results of a consistent comparison for 5-gene model (Supernatant) and 7 gene model (Sediment). [file 40169_2020_257_MOESM1_ESM.docx]

**Table S1. Primers list**

| FGFR2-549-U | AGAGATGGAGATGATGAAGATGATTGGGAA |
| --- | --- |
| FGFR2-549-D | AGTTTTTCCTCCTACTCACCATCCTGTGTG |
| HRAS-13-U | AGGCCCCTGAGGAGCGATGACGGAATATAA |
| HRAS-13-D | CACAAAATGGTTCTGGATCAGCTGGATGGT |
| KRAS-61-U | AAGTAGTAATTGATGGAGAAACCTGTCTCT |
| KRAS-61-D | CTCCCCAGTCCTCATGTACTGGTCCCTCAT |
| KRAS-13-U | ATAAGGCCTGCTGAAAATGACTGAATATAA |
| KRAS-13-D | TCCACAAAATGATTCTGAATTAGCTGTATC |
| ERBB3-104-U | TATGTCCTCGTGGCCATGAATGAATTCTCT |
| ERBB3-104-D | TGACGAAGATGGCAAACTTCCCATCGTAGA |
| ERBB3-297-U | TCATCTCTAATGGTGTCCTCCTCCTCTTCC |
| ERBB3-297-D | CCATCTTGTCAGGAGGACAGGCCCTGACAC |
| RB1-552-U | TTATCAAAGCAGAAGGCAACTTGACAAGAG |
| RB1-552-D | CTGAGAGCCATGCAAGGGATTCCATGATTC |
| BUB1B-705-U | CCTCGGTTGCAAGCACCTCCTCCATCAAAT |
| BUB1B-705-D | GGTATGTATATCCTACCTGAAGTCTCATTA |
| MAP2K1-124-U | GCAATCCGGAACCAGATCATAAGGGAGCTG |
| MAP2K1-124-D | TCGCCATCGCTGTAGAACGCACCATAGAAG |
| TP53-331-U | CTCCTCTCCCCAGCCAAAGAAGAAACCACT |
| TP53-331-D | CTGGAAACTTTCCACTTGATAAGAGGTCCC |
| TP53-306-U | GGAGAGACCGGCGCACAGAGGAAGAGAATC |
| TP53-306-D | TAACTGCACCCTTGGTCTCCTCCACCGCTT |
| TP53-249-U | CATCTTGGGCCTGTGTTATCTCCTAGGTTG |
| TP53-249-D | GCTCCTGACCTGGAGTCTTCCAGTGTGATG |
| TP53-220-U | GAAGGAAATTTGCGTGTGGAGTATTTGGAT |
| TP53-220-D | CTTAACCCCTCCTCCCAGAGACCCCAGTTG |
| TP53-205-U | GGGTCCCCAGGCCTCTGATTCCTCACTGAT |
| TP53-205-D | GGCACCACCACACTATGTCGAAAAGTGTTT |
| ZNF814--U | GAGAGTTCACACTGGGAAAAGACCTTATGA |
| ZNF814--D | GTGAACTCTCTGATGATTACTGAAGCTAGC |
| mTOR-2419-U | TCACTGTCCATCAGCCTCCAGTTCAGCAAG |
| mTOR-2419-D | ACAGTGATGGAGGTGCTGCGAGAGCACAAG |
| mTOR-2014-U | AGGCCTTCATGCCACATCTCATGCCAGAGG |
| mTOR-2014-D | GTTAGCTGATTCTTGATGCTTCTCTCCTTC |
| NRAS-61-U | TGGTTATAGATGGTGAAACCTGTTTGTTGG |
| NRAS-61-D | GCCTGTCCTCATGTATTGGTCTCTCATGGC |
| NRAS-13-U | GGTTCTTGCTGGTGTGAAATGACTGAGTAC |
| NRAS-13-D | ACAAAGTGGTTCTGGATTAGCTGGATTGTC |
| ARID1A-633-U | CTCAATGACCTCCAGTAAGGGAGGGCAAGA |
| ARID1A-633-D | TCAGAAGGTGCAGAAATACTCACAGGCAAG |
| U2AF1-34-U | CAACTGTTCATTTTATTTCAAAATTGGAGC |
| U2AF1-34-D | TGAAAAAAAGGCAAACAAACCTGGCTAAAC |
| EP300-65-U | TCTACAGAATTGGGACTAACCAATGGTGGT |
| EP300-65-D | CAGCTGTTTATGTTTAGAAGCTGCATCTTG |
| NFE2L2-79-U | CAAAAGGAGCAAGAGAAAGCCTTTTTCGCT |
| NFE2L2-79-D | GGATGTGCTGGGCTGGCTGAATTGGGAGAA |
| NFE2L2-34-U | TAGGACATGGATTTGATTGACATACTTTGG |
| NFE2L2-34-D | CAGCTCATACTCTTTCCGTCGCTGACTGAA |
| SF3B1-902-U | CATAAACTTGAAGAACAACTGATTGATGGT |
| SF3B1-902-D | TAGTAGCAATGTGCCATAATAGTTTTCATT |
| IDH1-132-U | ATATCCCCCGGCTTGTGAGTGGATGGGTAA |
| IDH1-132-D | CACATTATTGCCAACATGACTTACTTGATC |
| EIF2AK3-536-U | ACTGGTGGAAAGAAATAGTTGCAACGATTT |
| EIF2AK3-536-D | TACCCTGTGAGGATGAGGATGGAAAAGCCT |
| PIK3CB-1051-U | AGTGAAGAAGAAGCACTCAAACAGTTTAAG |
| PIK3CB-1051-D | GAACTGTGTGGGCCATCCAGTTCACTTTAG |
| PIK3CA-88-U | GTTACTCAAGAAGCAGAAAGGGAAGAATTT |
| PIK3CA-88-D | CTGGTTCAATTACTTTTAAAAAGGGTTGAA |
| PIK3CA-542-U | GACAAAGAACAGCTCAAAGCAATTTCTACA |
| PIK3CA-542-D | CCATTTTAGCACTTACCTGTGACTCCATAG |
| PIK3CA-1043-U | AGATAAAACTGAGCAAGAGGCTTTGGAGTA |
| PIK3CA-1043-D | TGTTTAATTGTGTGGAAGATCCAATCCATT |
| CTNNB1-32-U | GACAGAAAAGCGGCTGTTAGTCACTGGCAG |
| CTNNB1-32-D | GTATCCACATCCTCTTCCTCAGGATTGCCT |
| FBXW7-505-U | ATATTGAGACAGGCCAGTGTTTACATGTTT |
| FBXW7-505-D | TCATATGCTCCACTAACAACCCTCCTGCCA |
| FBXW7-465-U | ATGCAGAGACTGGAGAATGTATACACACCT |
| FBXW7-465-D | CCAACCATGACAAGATTTTCCCTTACCTTT |
| APC-1645-U | GTTAGTTTTACACCGGGGGATGATATGCCA |
| APC-1645-D | TGTTAGATCACTTAGAGATGTAGCTGTGGA |
| PRKAA1-246-U | GTGCCAACTCTTTTTAAGAAGATATGTGAT |
| PRKAA1-246-D | GCAGCATATGTTTCAAAAGGCTAATCACAG |
| BRAF-600-U | ACACCTCAGATATATTTCTTCATGAAGACC |
| BRAF-600-D | AGACAACTGTTCAAACTGATGGGACCCACT |
| BRAF-469-U | AGATTCCTGATGGGCAGATTACAGTGGGAC |
| BRAF-469-D | CAATGTCACCACATTACATACTTACCATGC |
| TSC1-636-U | TCAGGAAGACTGAGGAGCTGTTAAAGAAAG |
| TSC1-636-D | GTCCAGCACTTCCATTGGGGAGGTAGAGGG |
| STAG2-593-U | ATTTCACAGTACTCTGTAGATGCAGAAAAG |
| STAG2-593-D | TCTTACCTTTTCTAATCGTCCAGTGGTATA |
| STAG2-1029-U | AACAGTTTCGATTTCTTTTCAAGGTATGTT |
| STAG2-1029-D | AGACATCAGTGGAAGCCACACATCCTCTCT |
| ZRSR2-93-U | CTAAGAGAGCAGAAGGCACAAGAAGAATTC |
| ZRSR2-93-D | GTGATTCCTACCATACCTCTTGTTCTTCTT |
| KDM6A-863-U | CACAGTGGGCTACACACAATTAATGGAGAA |
| KDM6A-863-D | GGACTAGGTTTGTGGTTAACCAGAAGCAGA |
| KDM6A-1358-U | TGTCACTAATGAGAGTAATTCACGAAAGAC |
| KDM6A-1358-D | CAAAGTTTTCCAAGTTTCCGCTTGTTTTTC |
| RB1-217-U | TCAGGGGAAGTATTACAAATGGAAGATGAT |
| RB1-217-D | GTGAGAGTTTAATAAAATAGTCAAGGACAC |
| PIK3CA-345-U | TGGGTTATAAATAGTGCACTCAGAATAAAA |
| PIK3CA-345-D | TAATAAGCATCAGCATTTGACTTTACCTTA |
| STAG2--U | GTCATTTCACTTCTTACAGGATTGTCTGAC |
| STAG2--D | AAAATATTTCTAAATACCGACCTGCCAGGG |
| STAG2-1012-U | TTGGCATTTCTTGATATTCTGAGTGAATTT |
| STAG2-1012-D | AATAGGACATTTCCAGCAAATACATACACT |
| HRAS-61-U | GTGGTCATTGATGGGGAGACGTGCCTGTTG |
| HRAS-61-D | CTCCCCGGTGCGCATGTACTGGTCCCGCAT |
| RB1-850-U | AATTCTGTAATTTGTAGACTTCTGAGAAGT |
| RB1-850-D | TTCCTTCAGCACTTCTTTTGAGCACACGGT |
| AKT1-49-U | AATGATGGCACCTTCATTGGCTACAAGGAG |
| AKT1-49-D | GAGGCCAAGGGGATACTTACGCGCCACAGA |
| AKT1-17-U | ACGGGTAGAGTGTGCGTGGCTCTCACCACC |
| AKT1-17-D | ATTCTTGAGGAGGAAGTAGCGTGGCCGCCA |
| FOXA1-226-U | CAGCAGCGCTGGCAGAACTCCATCCGCCAC |
| FOXA1-226-D | CCCTTGCCCGGCTTGTCCGGGGAGCGTGCC |
| WASH3P--U | CTCCGGTGGCCGGGCCACTCTGCTAGAGTC |
| WASH3P--D | TGCTTCTTCTCCAGCTTTCGCTCCTTCATG |
| ERBB2-310-U | TGGCTACATGTTCCTGATCTCCTTAGACAA |
| ERBB2-310-D | ATCCTCTGCTGTCACCTCTTGGTTGTGCAG |
| ERBB2-733-U | GAGACGGAGCTGAGGAAGGTGAAGGTGCTT |
| ERBB2-733-D | TCCTCTGGGGCCGCCCACCCCAGGACCTGG |
| TP53-342-U | CCTCTGTTGCTGCAGATCCGTGGGCGTGAG |
| TP53-342-D | CCCAGCCTGGGCATCCTTGAGTTCCAAGGC |
| TP53-286-U | ACTGCCTCTTGCTTCTCTTTTCCTATCCTG |
| TP53-286-D | GCAGCTCGTGGTGAGGCTCCCCTTTCTTGC |
| TP53-179-U | GTCCGCGCCATGGCCATCTACAAGCAGTCA |
| TP53-179-D | CGTCTCTCCAGCCCCAGCTGCTCACCATCG |
| TP53-163-U | TTGCCAACTGGCCAAGACCTGCCCTGTGCA |
| TP53-163-D | GGGCAGCGCCTCACAACCTCCGTCATGTGC |
| TP53-141-U | GACTTTCAACTCTGTCTCCTTCCTCTTCCT |
| TP53-141-D | GTGCCGGGCGGGGGTGTGGAATCAACCCAC |
| ELF3-331-U | CTATGGGGCCAAAAGAAAAAGAACAGCAAC |
| ELF3-331-D | CATGTCCGGCTGTATCGTGAGGGTCCTGGC |
| ARID1A-566-U | TACTCACAGCCACAGGCTCAGTCTCCTTAC |
| ARID1A-566-D | AGGATACGCAGCCTGCTGGGAGAGCGTCGA |
| NCOA6--U | CAGCTAATTTTCCCCAGCTGCAGCAGCAGC |
| NCOA6--D | CTGTTGTTGCTGCTGTTGCTGTTGTTGCTG |
| FGFR3-370-U | CTCAACGCCCATGTCTTTGCAGCCGAGGAG |
| FGFR3-370-D | GAGCCCAGGCCTTTCTTGGGGGGGCTGCGC |
| CDKN1A-49-U | CGCGACTGTGATGCGCTAATGGCGGGCTGC |
| CDKN1A-49-D | AAGTCACCCTCCAGTGGTGTCTCGGTGACA |
| CUL1-485-U | ATGCTCGCCAAGAGGCTCGTCCACCAGAAC |
| CUL1-485-D | TTTTCAGGAAAAGATGAAACTCACCTTTAA |
| ACTB-348-U | GCTCCTCCTGAGCGCAAGTACTCCGTGTGG |
| ACTB-348-D | GTCATACTCCTGCTTGCTGATCCACATCTG |
| ACTB-158-U | GTACGCCTCTGGCCGTACCACTGGCATCGT |
| ACTB-158-D | ATGGGGGAGGGCATACCCCTCGTAGATGGG |
| CDKN2A-108-U | ACGCTGGTGGTGCTGCACCGGGCCG |
| CDKN2A-108-D | CATCGCGATGGCCCAGCTCCTCAGCCAGGT |
| CDKN2A-80-U | TGCTGCTCCACGGCGCGGAGCCCAACTGCG |
| CDKN2A-80-D | CACCACCAGCGTGTCCAGGAAGCCCTCCCG |
| CDKN2A-58-U | CCCTGGCTCTGACCATTCTGTTCTCTCTGG |
| CDKN2A-58-D | AGTTGGGCTCCGCGCCGTGGAGCAGCAGCA |
| KDM6A-555-U | GGCCAGCAGCCACAGCTTGCTCTGACCAGA |
| KDM6A-555-D | GGTCCATTGGCCAAAGGCTGCCCAGGGCAG |
| BRWD1-584-U | AGAGATTCTAATAATTATGTCTTAGATGAGC |
| BRWD1-584-D | GGATGAGGATTTCCATCTACATCTACCAAGA |
| RB1-765-U | ATGATTCTATTATAGTATTCTATAACTCGGTC |
| RB1-765-D | AAAGGATACTTTTGACCTACCCTGGTGGAAGC |
| FGFR3-650-U | AGATCGCAGACTTCGGGCTGGCCCG |
| FGFR3-650-D | ATGACCCCCACCCCCGCACCCC |
| FGFR3-248-U | TGAGGGAGGGGGTGGCCCCTGA |
| FGFR3-248-D | CGGCAGCCCCGCCTGCAGGATG |
| PTEN-233-U | CTGTTTGTGGAAGAACTCTACTTTGATATC |
| PTEN-233-D | GGTCTGCCAGCTAAAGGTGAAGATATATTC |
| ATM-158-U | ACAACAACCTTCAAAACATACCTAACCACT |
| ATM-158-D | GCAACATACTACTCAAAGACATTCTTTCTG |
| ATM-3007-U | GCCTTCTTCCACTCCTTTCAGTTTCTCTTG |
| ATM-3007-D | GTGTTTTTGTCCTTAGTGATATTGACCAGA |
| RB1-62-U | AAGTTAACCAAGCTCTCTCTCTGACATGAT |
| RB1-62-D | GGTAGGCTTGAGTTTGAAGAAACAGAAGAA |
| IDH2-48-U | GCTGTGTGTCCCTGCTTCTAGATGCCGACA |
| IDH2-48-D | GAACTGCCAGATAATACGGGTCATCTCATC |
| ERBB2-769-U | GGGGTCCTTCCTGTCCTCCTAGCAGGAGAG |
| ERBB2-769-D | TTGAGGGAAAACACATCCCCCAAAGCCAAC |
| PIK3CA-451-U | TACTTTATTTGGATTTGATCCAGTAACACC |
| PIK3CA-451-D | CTCTAGTATCTGGAAAAATGGCTTTGAATC |
| BRAF-695-U | CAGTAAGGTACGGAGTAACTGTCCAAAAGC |
| BRAF-695-D | ACTTACTTGGGGAAAGAGTGGTCTCTCATC |
| BRAF-595-U | ACACCTCAGATATATTTCTTCATGAAGACC |
| BRAF-595-D | ACTGATGGGACCCACTCCATCGAGATTTCA |
| BRAF-446-U | GGCTTGACTTGACTTTTTTACTGTTTTTAT |
| BRAF-446-D | TTTGTCCCACTGTAATCTGCCCATCAGGAA |
| EGFR-293-U | CAAGGATGCCTGACCAGTTAGAGGGCCCAC |
| EGFR-293-D | GAACCCCGAGGGCAAATACAGCTTTGGTGC |
| EGFR-384-U | TGATTTCCTTTACGGTTTTCAGAATATCCA |
| EGFR-384-D | ATAATCACCCTGTTGTTTGTTTCAGTGACT |
| EGFR-836-U | AAATCTGTGATCTTGACATGCTGCGGTGTT |
| EGFR-836-D | CTGTTTCAGGGCATGAACTACTTGGAGGAC |
| EGFR-859-U | AGCCTGGTCCCTGGTGTCAGGAAAATGCTG |
| EGFR-859-D | GTGAAAACACCGCAGCATGTCAAGATCACA |
| TSC1-328-U | TACTCCACGTCTCGGCTGATGTTGTTAAAT |
| TSC1-328-D | TACTTGTGGTGGTTCAGTTATCAGCCGTGT |
| TSC1-190-U | AAGTCTATCTCGTCCATCTCCATGCCAGTG |
| TSC1-190-D | ACGCAAAAAGGAGACGAAGTTGCAAGGGTA |
| ATM-982-U | ACTACATGAAGGACATGGTTTAAAATAGTT |
| ATM-982-D | TTTTTTTTTTTTTACCACAGCAATGTGTGT |
| RB1-567-U | GATCAGTTGGTCCTTCTCGGTCCTTTGATT |
| RB1-567-D | GGGAAAATTATGCTTACTAATGTGGTTTTA |
| ERBB2-313-U | CCGCTGTGTTCCATCCTCTGCTGTCACCTC |
| ERBB2-313-D | TCCTTAGACAACTACCTTTCTACGGACGTG |
| IDH1-320-U | TAGAAGCAGAGGCTGCCCACGGGACTGTAA |
| IDH1-320-D | AACACCATCTTACCAATGGGATTGGTGGAC |
| PIK3CA-365-U | TGGAACAAGGTACTCTTTGAGTGTTCACAT |
| PIK3CA-365-D | TTCTTTTAGATCTATGTTCGAACAGGTATC |
| IDH2-208-U | ACTTTCAAAATGGTCTTCACCCCAAAAGAT |
| IDH2-208-D | TACATGCCCATGCCCACGCCGCCTGCGGGG |
| TSC2-367-U | TCCCCGGAGCTCCTGCCCCCACCCCACCTG |
| TSC2-367-D | CTCCAGGTGGTGGCGTGGGACATTCTGCTG |
| TSC2-656-U | CAGGAGGCCCTGTGGGAGGAGAAAGGGGGC |
| TSC2-656-D | GGCCTCAGCTGCTTCTCTTGCTTCTGCAGG |
| CCNE1-95-U | GACCTCAGTACAGGCAGCGGGGAGCCTCTG |
| CCNE1-95-D | CCTGACAAAGAAGATGATGACCGGGTTTAC |
| FGFR3-216-U | CTGCCGGATGCTGCCAAACTTGTTCTCCAC |
| FGFR3-216-D | GTCCCGGTGCAGCTGCGGCATCAGCAGTGG |
| CDKN1A-48-U | GAGCTTGGGCAGGCCAAGGCCCCGCACACG |
| CDKN1A-48-D | AGCCGCGACTGTGATGCGCTAATGGCGGGC |
| EGFR--U | TCCCGCCACTGGATGCTCTCCACGTTGCAC |
| EGFR--D | TTAAAGGAGCTGGAAAGAGTGCTCACCGCA |
| EGFR-627-U | CTCTGAATTTGCAAGAGAGGAAATGTTCTG |
| EGFR-627-D | CGCAGACGCCGGCCATGTGTGCCACCTGTG |
| EGFR-1079-U | AAGCCACTCACCAGGCACTGGGAGGAAGGT |
| EGFR-1079-D | GCTTCTTGCAGCGATACAGCTCAGACCCCA |
| EGFR-1118-U | TTGCCCACTGCAGTGCTGTGGGGGTCCTGG |
| EGFR-1118-D | TGGCTCTGTGCAGAATCCTGTCTATCACAA |
| TSC1-871-U | CTTGGGGAGGTCAACGAGCTCTATTTGGAA |
| TSC1-871-D | TAAGGTCTGGCTCCCGAGCCCTGGCATACC |
| TSC1-536-U | CACTCGGCAGCCTCCAGTTCTCAGGGCGCC |
| TSC1-536-D | GTAAAGGCTTGCTTTGGTGTGTCAGGCCCA |
| TSC1-516-U | GTTCCTCGAGGAGGCTTTGACTCTCCCTTT |
| TSC1-516-D | ACGCTGGCGCCCTGAGAACTGGAGGCTGCC |
| ATM-337-U | ACAGATATCTGCCATCAATTCAATCAAATT |
| ATM-337-D | GATAAGTCATATAGGAAGTAGAGGAAAGTA |
| ATM-2815-U | CTGGTTGAAAATTTTGGCAAACATCCATGA |
| ATM-2815-D | TATATTCTCTATTTAAAGGAGGTGCAAAAA |
| RB1-780-U | AAGGGTGAACTAGGAAACTTGTAAGGGCTT |
| RB1-780-D | AATAATCTACTTTTTTGTTTTTGCTCTAGC |
| PIK3CA-417-U | GTTTTTAAAGGTAAACATAATTGTTCCTTC |
| PIK3CA-417-D | CTTTGCCTTTCCATTTGCTCTGTTAAAGGC |
| ATM-2888-U | ACCAAATATTTACTAAAAAGAATAGATACATAGAT |
| ATM-2888-D | ACAGAATATCTTGATAAATGAGCAGTCAGC |
| RB1-844-U | TTCTTTTGAGCACACGGTCGCTGTTACATA |
| RB1-844-D | GCATTTAATGATTTAAAGTAAAGAATTCTGT |
| TERT-228-U | ACCTTCCAGCTCCGCCTCCTCCGCGCGGAC |
| TERT-228-D | AGAGGGCGGGGCCGCGGAAAGGAAGGGGAG |

**Table S2. Average of unique reads for each sample.**

| **Sample ID** | **Average depth** |
| --- | --- |
| KYBC0058-plasma | 790.07 |
| KYBC0004-plasma | 866.75 |
| KYBC0008-plasma | 1043.05 |
| KYBC0063-plasma | 1066.37 |
| KYBC0019-plasma | 1082.26 |
| KYBC0090-plasma | 1161.83 |
| KYBC0009-plasma | 1207.77 |
| KYBC0040-plasma | 1243.96 |
| KYBC0025-plasma | 1349.33 |
| KYBC0012-plasma | 1562.08 |
| KYBC0049-plasma | 1600.87 |
| KYBC0046-plasma | 1793.74 |
| KYBC0087-plasma | 2498.31 |
| KYBC0071-plasma | 2615.93 |
| KYBC0033-plasma | 3272.52 |
| KYBC0053-plasma | 3530.23 |
| KYBC0074-sediment | 166.47 |
| KYBC0042-sediment | 338.81 |
| KYBC0043-sediment | 361.84 |
| KYBC0087-sediment | 466.72 |
| KYBC0014-sediment | 547.73 |
| KYBC0078-sediment | 890.75 |
| KYBC0038-sediment | 901.09 |
| KYBC0224-sediment | 1022 |
| KYBC0225-sediment | 1027 |
| KYBC0214-sediment | 1054 |
| KYBC0109-sediment | 1202 |
| KYBC0235-sediment | 1202 |
| KYBC0215-sediment | 1206 |
| KYBC0237-sediment | 1206 |
| KYBC0253-sediment | 1255 |
| KYBC0217-sediment | 1261 |
| KYBC0254-sediment | 1261 |
| KYBC0255-sediment | 1262 |
| KYBC0238-sediment | 1263 |
| KYBC0239-sediment | 1264 |
| KYBC0240-sediment | 1266 |
| KYBC0233-sediment | 1272 |
| KYBC0204-sediment | 1277 |
| KYBC0241-sediment | 1277 |
| KYBC0008-sediment | 1327.08 |
| KYBC0101-sediment | 1435.06 |
| KYBC0072-sediment | 1444.95 |
| KYBC0028-sediment | 1561.97 |
| KYBC0029-sediment | 1666.62 |
| KYBC0002-sediment | 1695.7 |
| KYBC0071-sediment | 1705.56 |
| KYBC0086-sediment | 1726.36 |
| KYBC0013-sediment | 1841.13 |
| KYBC0033-sediment | 1843.01 |
| KYBC0005-sediment | 1869.78 |
| KYBC0023-sediment | 1890.34 |
| KYBC0218-sediment | 1920 |
| KYBC0089-sediment | 1933.92 |
| KYBC0205-sediment | 1983 |
| KYBC0242-sediment | 1983 |
| KYBC0206-sediment | 1984 |
| KYBC0243-sediment | 1984 |
| KYBC0207-sediment | 1991 |
| KYBC0216-sediment | 1991 |
| KYBC0227-sediment | 1991 |
| KYBC0244-sediment | 1991 |
| KYBC0208-sediment | 2005 |
| KYBC0245-sediment | 2005 |
| KYBC0219-sediment | 2008 |
| KYBC0228-sediment | 2142 |
| KYBC0220-sediment | 2174 |
| KYBC0123-sediment | 2183 |
| KYBC0186-sediment | 2223 |
| KYBC0234-sediment | 2225 |
| KYBC0229-sediment | 2262 |
| KYBC0202-sediment | 2268 |
| KYBC0200-sediment | 2294 |
| KYBC0201-sediment | 2297 |
| KYBC0088-sediment | 2347.91 |
| KYBC0083-sediment | 2350.56 |
| KYBC0230-sediment | 2405 |
| KYBC0009-sediment | 2439.75 |
| KYBC0004-sediment | 2441.67 |
| KYBC0090-sediment | 2457.74 |
| KYBC0231-sediment | 2479 |
| KYBC0032-sediment | 2489.13 |
| KYBC0006-sediment | 2522.85 |
| KYBC0016-sediment | 2568.27 |
| KYBC0040-sediment | 2574.74 |
| KYBC0212-sediment | 2606 |
| KYBC0221-sediment | 2606 |
| KYBC0031-sediment | 2665.66 |
| KYBC0025-sediment | 2684.8 |
| KYBC0053-sediment | 2713.2 |
| KYBC0019-sediment | 2714.65 |
| KYBC0111-sediment | 2766.79 |
| KYBC0082-sediment | 2815.52 |
| KYBC0076-sediment | 2876.63 |
| KYBC0112-sediment | 2879.43 |
| KYBC0012-sediment | 2896.58 |
| KYBC0187-sediment | 2906 |
| KYBC0034-sediment | 2920.84 |
| KYBC0190-sediment | 2941 |
| KYBC0211-sediment | 2980 |
| KYBC0248-sediment | 2980 |
| KYBC0249-sediment | 2985 |
| KYBC0010-sediment | 3001.97 |
| KYBC0250-sediment | 3005 |
| KYBC0251-sediment | 3006 |
| KYBC0098-sediment | 3012.4 |
| KYBC0252-sediment | 3013 |
| KYBC0037-sediment | 3013.13 |
| KYBC0011-sediment | 3090.69 |
| KYBC0110-sediment | 3222.13 |
| KYBC0058-sediment | 3363.51 |
| KYBC0041-sediment | 3417.82 |
| KYBC0191-sediment | 3430 |
| KYBC0055-sediment | 3450.69 |
| KYBC0091-sediment | 3523.15 |
| KYBC0192-sediment | 3528 |
| KYBC0197-sediment | 3534 |
| KYBC0063-sediment | 3618.96 |
| KYBC0095-sediment | 3649.67 |
| KYBC0108-sediment | 3662.72 |
| KYBC0051-sediment | 3742.33 |
| KYBC0049-sediment | 3771.49 |
| KYBC0060-sediment | 3871.11 |
| KYBC0046-sediment | 3904.06 |
| KYBC0232-sediment | 4338 |
| KYBC0213-sediment | 4354 |
| KYBC0223-sediment | 4467 |
| KYBC0115-sediment | 4488 |
| KYBC0122-sediment | 4741 |
| KYBC0203-sediment | 4908 |
| KYBC0199-sediment | 4932 |
| KYBC0165-sediment | 5050 |
| KYBC0179-sediment | 5058 |
| KYBC0183-sediment | 5114 |
| KYBC0209-sediment | 5190 |
| KYBC0246-sediment | 5190 |
| KYBC0210-sediment | 5198 |
| KYBC0247-sediment | 5198 |
| KYBC0226-sediment | 5799 |
| KYBC0198-sediment | 7143 |
| KYBC0222-sediment | 7210 |
| KYBC0016-supernatant | 189.77 |
| KYBC0009-supernatant | 328.66 |
| KYBC0028-supernatant | 359.97 |
| KYBC0072-supernatant | 468.34 |
| KYBC0074-supernatant | 480.46 |
| KYBC0055-supernatant | 497.04 |
| KYBC0063-supernatant | 515.85 |
| KYBC0029-supernatant | 592.22 |
| KYBC0095-supernatant | 636.43 |
| KYBC0086-supernatant | 637.71 |
| KYBC0013-supernatant | 725.29 |
| KYBC0042-supernatant | 737.2 |
| KYBC0014-supernatant | 773.44 |
| KYBC0040-supernatant | 784.76 |
| KYBC0008-supernatant | 793.75 |
| KYBC0012-supernatant | 809.68 |
| KYBC0006-supernatant | 893.94 |
| KYBC0038-supernatant | 987.77 |
| KYBC0060-supernatant | 1025.58 |
| KYBC0244-supernatant | 1027 |
| KYBC0051-supernatant | 1030.86 |
| KYBC0032-supernatant | 1048.16 |
| KYBC0190-supernatant | 1050 |
| KYBC0191-supernatant | 1054 |
| KYBC0201-supernatant | 1054 |
| KYBC0253-supernatant | 1054 |
| KYBC0202-supernatant | 1090 |
| KYBC0254-supernatant | 1090 |
| KYBC0076-supernatant | 1132.87 |
| KYBC0043-supernatant | 1152.5 |
| KYBC0041-supernatant | 1162.72 |
| KYBC0101-supernatant | 1208.85 |
| KYBC0212-supernatant | 1255 |
| KYBC0213-supernatant | 1261 |
| KYBC0214-supernatant | 1262 |
| KYBC0203-supernatant | 1272 |
| KYBC0255-supernatant | 1272 |
| KYBC0005-supernatant | 1353.87 |
| KYBC0031-supernatant | 1466.02 |
| KYBC0110-supernatant | 1466.04 |
| KYBC0049-supernatant | 1578.76 |
| KYBC0108-supernatant | 1579.08 |
| KYBC0090-supernatant | 1593.38 |
| KYBC0087-supernatant | 1611.77 |
| KYBC0023-supernatant | 1663.86 |
| KYBC0111-supernatant | 1675.97 |
| KYBC0082-supernatant | 1706.68 |
| KYBC0091-supernatant | 1835.46 |
| KYBC0083-supernatant | 1895.17 |
| KYBC0115-supernatant | 1903 |
| KYBC0221-supernatant | 1920 |
| KYBC0222-supernatant | 1930 |
| KYBC0223-supernatant | 1942 |
| KYBC0224-supernatant | 1967 |
| KYBC0225-supernatant | 1973 |
| KYBC0198-supernatant | 2005 |
| KYBC0226-supernatant | 2008 |
| KYBC0227-supernatant | 2017 |
| KYBC0122-supernatant | 2025 |
| KYBC0123-supernatant | 2171 |
| KYBC0204-supernatant | 2225 |
| KYBC0228-supernatant | 2250 |
| KYBC0229-supernatant | 2253 |
| KYBC0230-supernatant | 2262 |
| KYBC0231-supernatant | 2268 |
| KYBC0232-supernatant | 2394 |
| KYBC0165-supernatant | 2408 |
| KYBC0037-supernatant | 2414.51 |
| KYBC0109-supernatant | 2422 |
| KYBC0078-supernatant | 2428.76 |
| KYBC0233-supernatant | 2430 |
| KYBC0234-supernatant | 2479 |
| KYBC0058-supernatant | 2562.05 |
| KYBC0034-supernatant | 2577.37 |
| KYBC0205-supernatant | 2606 |
| KYBC0248-supernatant | 2606 |
| KYBC0004-supernatant | 2697.92 |
| KYBC0235-supernatant | 2941 |
| KYBC0207-supernatant | 2980 |
| KYBC0208-supernatant | 2985 |
| KYBC0209-supernatant | 3005 |
| KYBC0210-supernatant | 3006 |
| KYBC0211-supernatant | 3013 |
| KYBC0186-supernatant | 3016 |
| KYBC0187-supernatant | 3019 |
| KYBC0011-supernatant | 3169.76 |
| KYBC0215-supernatant | 3182 |
| KYBC0071-supernatant | 3280.81 |
| KYBC0112-supernatant | 3304.55 |
| KYBC0033-supernatant | 3347.26 |
| KYBC0219-supernatant | 3413 |
| KYBC0220-supernatant | 3416 |
| KYBC0237-supernatant | 3534 |
| KYBC0239-supernatant | 3640 |
| KYBC0241-supernatant | 3665 |
| KYBC0098-supernatant | 3726.52 |
| KYBC0010-supernatant | 3859.97 |
| KYBC0088-supernatant | 3893.23 |
| KYBC0002-supernatant | 4041.02 |
| KYBC0089-supernatant | 4058.62 |
| KYBC0046-supernatant | 4158.66 |
| KYBC0240-supernatant | 4327 |
| KYBC0206-supernatant | 4354 |
| KYBC0249-supernatant | 4354 |
| KYBC0242-supernatant | 4488 |
| KYBC0025-supernatant | 4588.53 |
| KYBC0053-supernatant | 4888 |
| KYBC0243-supernatant | 5027 |
| KYBC0192-supernatant | 5126 |
| KYBC0250-supernatant | 5126 |
| KYBC0216-supernatant | 5175 |
| KYBC0217-supernatant | 5181 |
| KYBC0218-supernatant | 5187 |
| KYBC0199-supernatant | 5190 |
| KYBC0197-supernatant | 5239 |
| KYBC0251-supernatant | 5239 |
| KYBC0019-supernatant | 5284.21 |
| KYBC0179-supernatant | 5736 |
| KYBC0245-supernatant | 5799 |
| KYBC0246-supernatant | 5802 |
| KYBC0247-supernatant | 5834 |
| KYBC0183-supernatant | 6091 |
| KYBC0252-supernatant | 6135 |
| KYBC0200-supernatant | 6342 |
| KYBC0238-supernatant | 7143 |
| KYBC0071-tissue | 417.9 |
| KYBC0063-tissue | 771.47 |
| KYBC0049-tissue | 846.86 |
| KYBC0087-tissue | 1231.75 |
| KYBC0058-tissue | 1272.25 |
| KYBC0033-tissue | 1494.85 |
| KYBC0046-tissue | 1790.35 |
| KYBC0090-tissue | 2039.04 |
| KYBC0009-tissue | 2191.26 |
| KYBC0004-tissue | 2675.08 |
| KYBC0025-tissue | 3072.78 |
| KYBC0008-tissue | 3100.63 |
| KYBC0040-tissue | 3128.77 |
| KYBC0012-tissue | 3236.98 |
| KYBC0019-tissue | 3786.64 |
| KYBC0053-tissue | 3810.11 |

**Table S3. The diagnostic parameters for the diagnostic model with increasing numbers of genes used .**

|  | | **Gene Number** | **AUC** | **Sensitivity** | **Specificity** | **PPV** | **NPV** |
| --- | --- | --- | --- | --- | --- | --- | --- |
| **Supernatant** | | 1 | 0.7283 | 0.4565 | 1.0000 | 1.0000 | 0.3976 |
|  |  | 2 | 0.8541 | 0.6957 | 1.0000 | 1.0000 | 0.5410 |
|  |  | 3 | 0.9269 | 0.8370 | 1.0000 | 1.0000 | 0.6875 |
|  |  | 4 | 0.9361 | 0.8587 | 1.0000 | 1.0000 | 0.7174 |
|  |  | 5 | 0.9401 | 0.8696 | 1.0000 | 1.0000 | 0.7333 |
|  |  | 6 | 0.9455 | 0.8804 | 1.0000 | 1.0000 | 0.7500 |
|  |  | 7 | 0.9489 | 0.8804 | 1.0000 | 1.0000 | 0.7500 |
|  |  | 8 | 0.8207 | 0.6413 | 1.0000 | 1.0000 | 0.5000 |
|  |  | 9 | 0.8261 | 0.6522 | 1.0000 | 1.0000 | 0.5077 |
|  |  | 10 | 0.9088 | 0.8478 | 0.9697 | 1.0000 | 0.7674 |
|  |  | 11 | 0.9359 | 0.9022 | 0.9697 | 0.9881 | 0.7805 |
|  |  | 12 | 0.4934 | 0.9565 | 0.0303 | 0.7333 | 0.2000 |
|  |  | 13 | 0.8424 | 0.6848 | 1.0000 | 1.0000 | 0.5323 |
|  |  | 14 | 0.8207 | 0.6413 | 1.0000 | 1.0000 | 0.5410 |
|  |  | 15 | 0.4934 | 0.9565 | 0.0303 | 0.7333 | 0.2000 |
|  |  | 16 | 0.8696 | 0.7391 | 1.0000 | 1.0000 | 0.4459 |
|  |  | 17 | 0.8261 | 0.6522 | 1.0000 | 1.0000 | 0.5156 |
|  |  | 18 | 0.8750 | 0.7500 | 1.0000 | 1.0000 | 0.6226 |
|  |  | 19 | 0.9572 | 0.8913 | 1.0000 | 1.0000 | 0.7674 |
| 1 | 0.7391 | 0.4783 | 1.0000 | 1.0000 | 0.4074 |  |  |
| 2 | 0.8169 | 0.6522 | 0.9697 | 0.9836 | 0.5000 |  |  |
| 3 | 0.8742 | 0.7500 | 0.9697 | 0.9857 | 0.5818 |  |  |
| 4 | 0.9002 | 0.8478 | 0.9091 | 0.9630 | 0.6818 |  |  |
| 5 | 0.8850 | 0.7826 | 0.9697 | 0.9639 | 0.7143 |  |  |
| 6 | 0.8982 | 0.8043 | 0.9697 | 0.9867 | 0.6400 |  |  |
| 7 | 0.9071 | 0.8261 | 0.9697 | 0.9870 | 0.6667 |  |  |
| 8 | 0.9160 | 0.8261 | 0.9697 | 0.9870 | 0.6667 |  |  |
| 9 | 0.9226 | 0.8370 | 0.9697 | 0.9872 | 0.6809 |  |  |
| 10 | 0.9226 | 0.8370 | 0.9697 | 0.9872 | 0.6809 |  |  |
| 11 | 0.9226 | 0.8370 | 0.9697 | 0.9872 | 0.6809 |  |  |
| 12 | 0.9226 | 0.8370 | 0.9697 | 0.9872 | 0.6809 |  |  |
| 13 | 0.9226 | 0.8370 | 0.9697 | 0.9872 | 0.6809 |  |  |
| 14 | 0.9130 | 0.8370 | 0.9697 | 0.9872 | 0.6809 |  |  |
| 15 | 0.9130 | 0.8370 | 0.9697 | 0.9872 | 0.6809 |  |  |
|  |  |  |  |  |  |  |  |

**Table S4. AUC values for each gene, in discriminating cancer from controls.**

|  | **Gene** | **AUC** | **CI** |
| --- | --- | --- | --- |
| **Supernatant** | TERT | 0.7283 | 0.6771-0.7794 |
|  | FGFR3 | 0.6805 | 0.6258-0.7352 |
|  | TP53 | 0.6467 | 0.6-0.6935 |
|  | PIK3CA | 0.6079 | 0.551-0.6647 |
|  | KRAS | 0.5815 | 0.5436-0.6195 |
|  | AKT1 | 0.538 | 0.5108-0.5653 |
|  | HRAS | 0.5351 | 0.4936-0.5765 |
|  | ERBB2 | 0.5326 | 0.5072-0.558 |
|  | U2AF1 | 0.5096 | 0.4782-0.5409 |
|  | ACTB | 0.5217 | 0.5008-0.5427 |
|  | CDKN2A | 0.5489 | 0.5184-0.5794 |
|  | KDM6A | 0.5016 | 0.4673-0.536 |
|  | CUL1 | 0.5054 | 0.4948-0.5161 |
|  | BRAF | 0.5163 | 0.4981-0.5346 |
|  | EGFR | 0.5109 | 0.4959-0.5259 |
|  | FBXW7 | 0.5054 | 0.4948-0.5161 |
|  | PTEN | 0.5054 | 0.4948-0.5161 |
|  | STAG2 | 0.5054 | 0.4948-0.5161 |
|  | CTNNB1 | 0.5054 | 0.4948-0.5161 |
| **Sediment** | TERT | 0.7391 | 0.6878-0.7904 |
|  | FGFR3 | 0.6657 | 0.6044-0.727 |
|  | TP53 | 0.5369 | 0.4643-0.6095 |
|  | HRAS | 0.5707 | 0.5349-0.6064 |
|  | PIK3CA | 0.6021 | 0.5472-0.6571 |
|  | KRAS | 0.5761 | 0.5392-0.613 |
|  | ERBB2 | 0.5326 | 0.5072-0.558 |
|  | AKT1 | 0.538 | 0.5108-0.5653 |
|  | KDM6A | 0.5109 | 0.4959-0.5259 |
|  | CDKN2A | 0.5489 | 0.5184-0.5794 |
|  | CTNNB1 | 0.5054 | 0.4948-0.5161 |
|  | BRAF | 0.5163 | 0.4981-0.5346 |
|  | FBXW7 | 0.5054 | 0.4948-0.5161 |
|  | PTEN | 0.5054 | 0.4948-0.5161 |
|  | STAG2 | 0.5054 | 0.4948-0.5161 |

**Table S5. Basic information of 12 false-negative samples.**

| **Layered** | **Group** | **Sample number** |
| --- | --- | --- |
| Gender | Male | 10 |
|  | Female | 2 |
| Age | <60 year | 3 |
|  | ≥60 year | 9 |
| Stage | <T2 | 9 |
|  | ≥T2 | 3 |

**Table S6. The results of a consistent comparison in different cancer stage.**

| **Group** | **Stage** | **Sensitivity** | **Sample number** | **p-value** |
| --- | --- | --- | --- | --- |
| Supernatant | <T2 | 0.8571 | 63 | 0.9460 |
|  | ≥T2 | 0.8889 | 27 |  |
| Sediment | <T2 | 0.8571 | 63 | 0.3064 |
|  | ≥T2 | 0.7407 | 27 |  |

**Table S7. The results of a consistent comparison in different gender.**

| **Group** | **Gender** | **Sensitivity** | **Sample number** | **p-value** |
| --- | --- | --- | --- | --- |
| Supernatant | Male  Female | 0.8718 | 78 | 1 |
|  |  | 0.8571 | 14 |  |
| Sediment | Male  Female | 0.8333 | 78 | 0.9601 |
|  |  | 0.7857 | 14 |  |

**Table S8. The results of a consistent comparison in different age.**

| **Group** | **Age** | **Sensitivity** | **Sample number** | **p-value** |
| --- | --- | --- | --- | --- |
| Supernatant | <60  ≥60 | 0.8750 | 24 | 1 |
|  |  | 0.8677 | 68 |  |
| Sediment | <60  ≥60 | 0.8750 | 24 | 0.6729 |
|  |  | 0.8088 | 68 |  |

**Table S9. The results of a consistent comparison for 5-gene model (Supernatant) and 7 gene model (Sediment).**

| **Group** | **Accuracy** | **Sample number** | **Sensitivity** | **Specificity** | **p-value** | **Kappa** |
| --- | --- | --- | --- | --- | --- | --- |
| Supernatant | 0.9040 | 125 | 0.8696 | 1 | 0.4295 | 0.6407 |
| Sediment | 0.8640 | 125 | 0.8261 | 0.9697 |  |  |
