# Supplementary figures and images for: Detection of bladder cancer using urinary cell-free DNA and cellular DNA
Source: Clin Transl Med. 2020 Jan 14;9:4. doi: 10.1186/s40169-020-0257-2 (PMC6960275; doi:10.1186/s40169-020-0257-2)

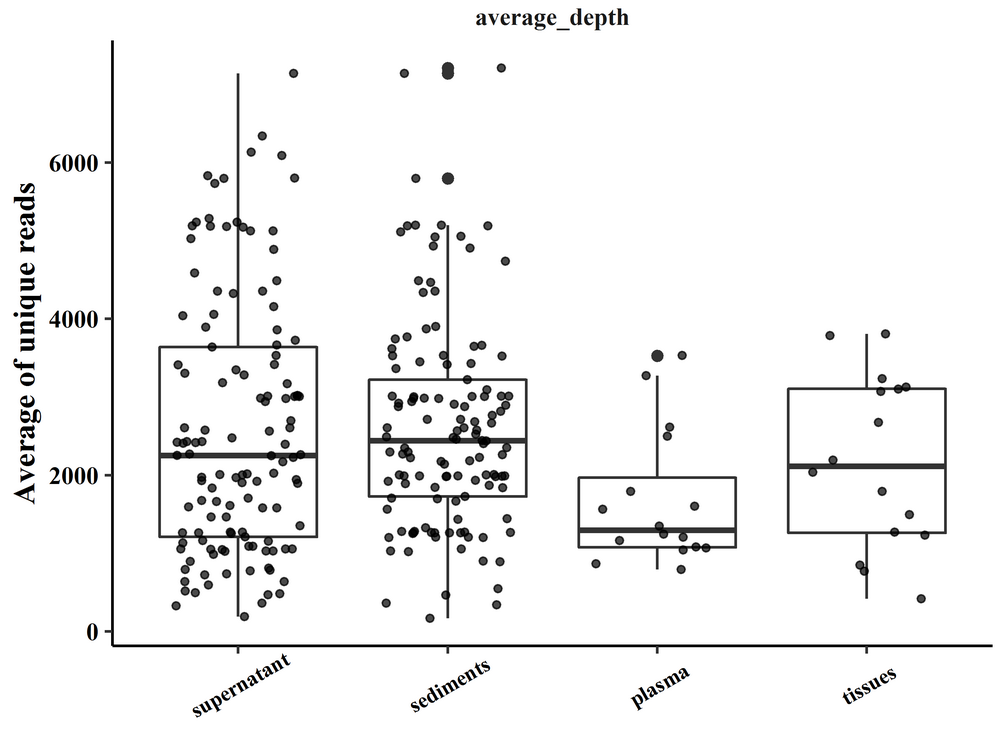

Supplement: Supplementary file 2 — Additional file 2: Figure S1. The average mutation depth of four types of samples. [file 40169_2020_257_MOESM2_ESM.tif]

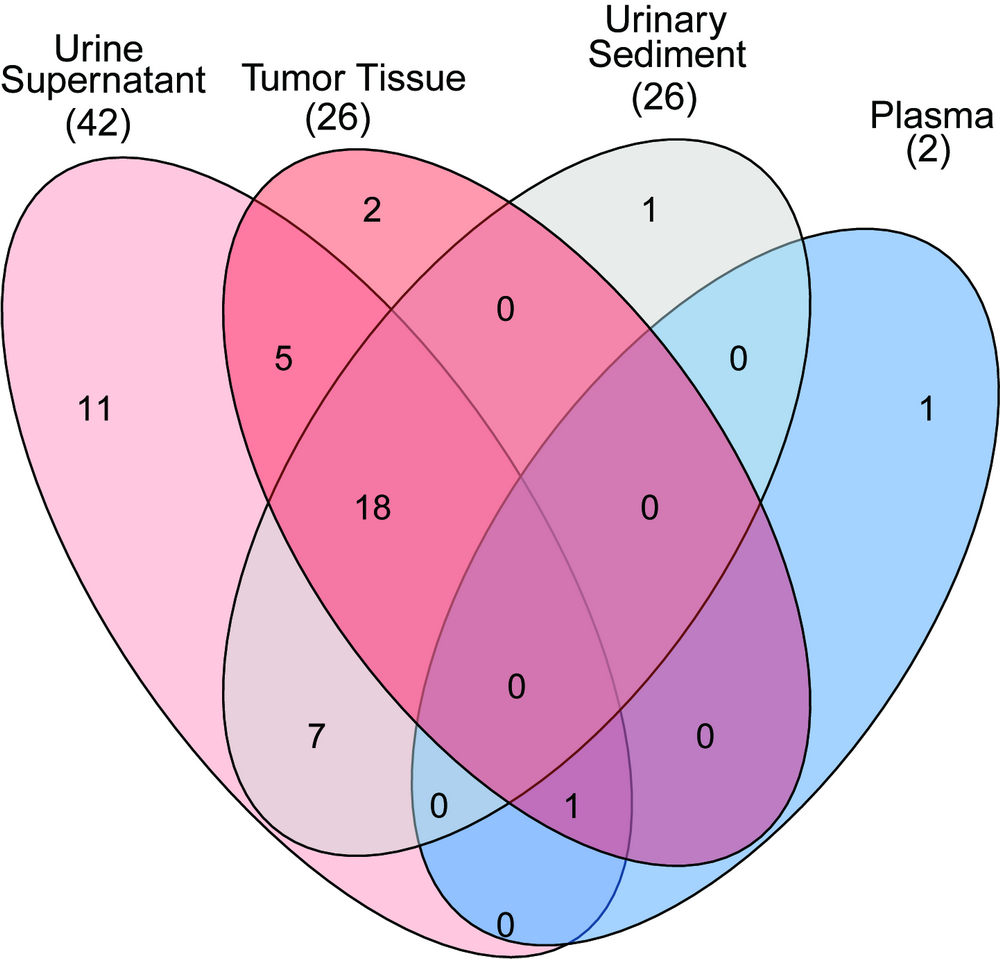

Supplement: Supplementary file 3 — Additional file 3: Figure S2. The mutations identified in urine supernatant, urine sediments, plasma, and cancer tissue. [file 40169_2020_257_MOESM3_ESM.tif]

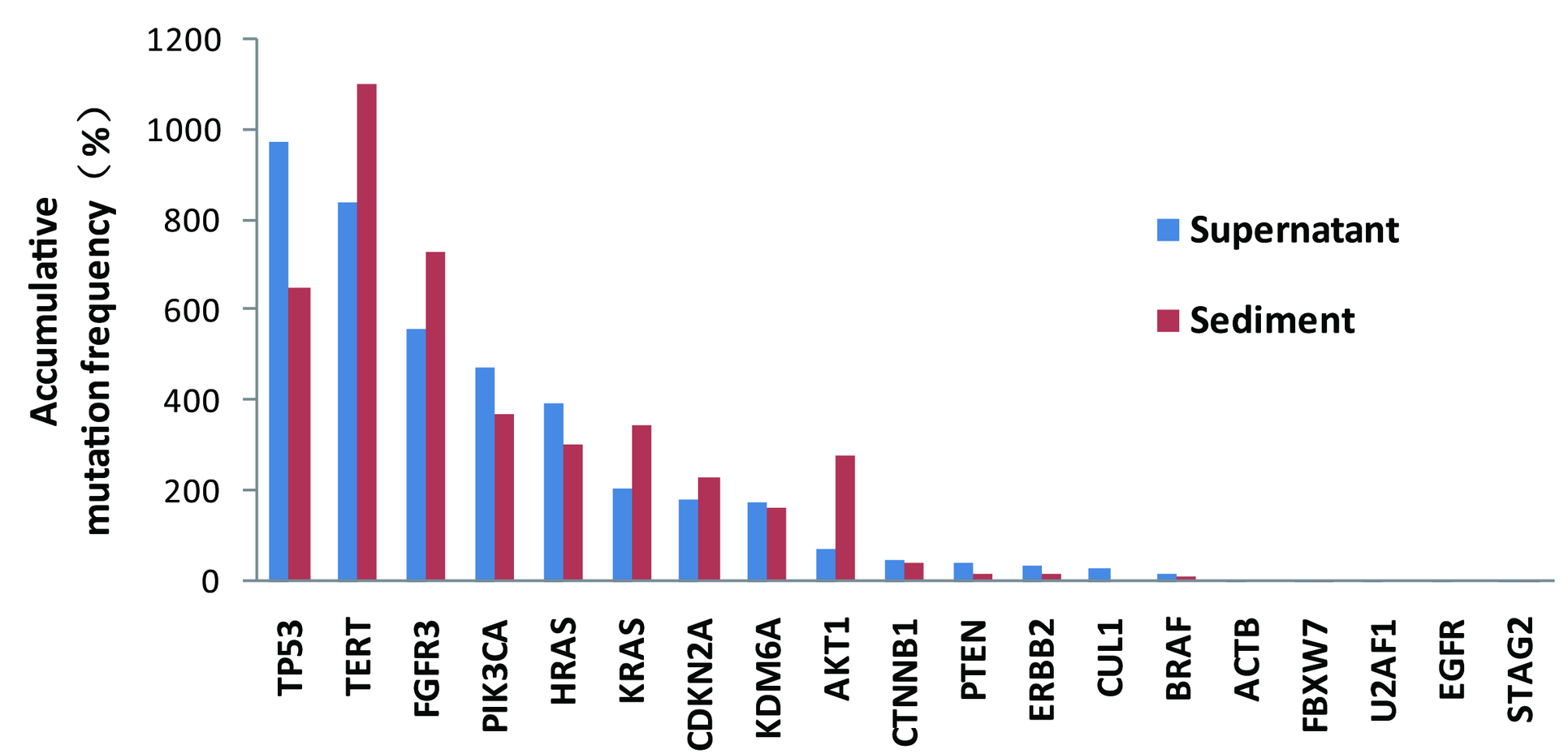

Supplement: Supplementary file 4 — Additional file 4: Figure S3. Cumulative mutation rates of DNA isolated from urine supernatant and sediments in 125 cases with hematuria. These mutations were noted in 19 and 15 genes, respectively, in urine supernatant and sediments. [file 40169_2020_257_MOESM4_ESM.tif]

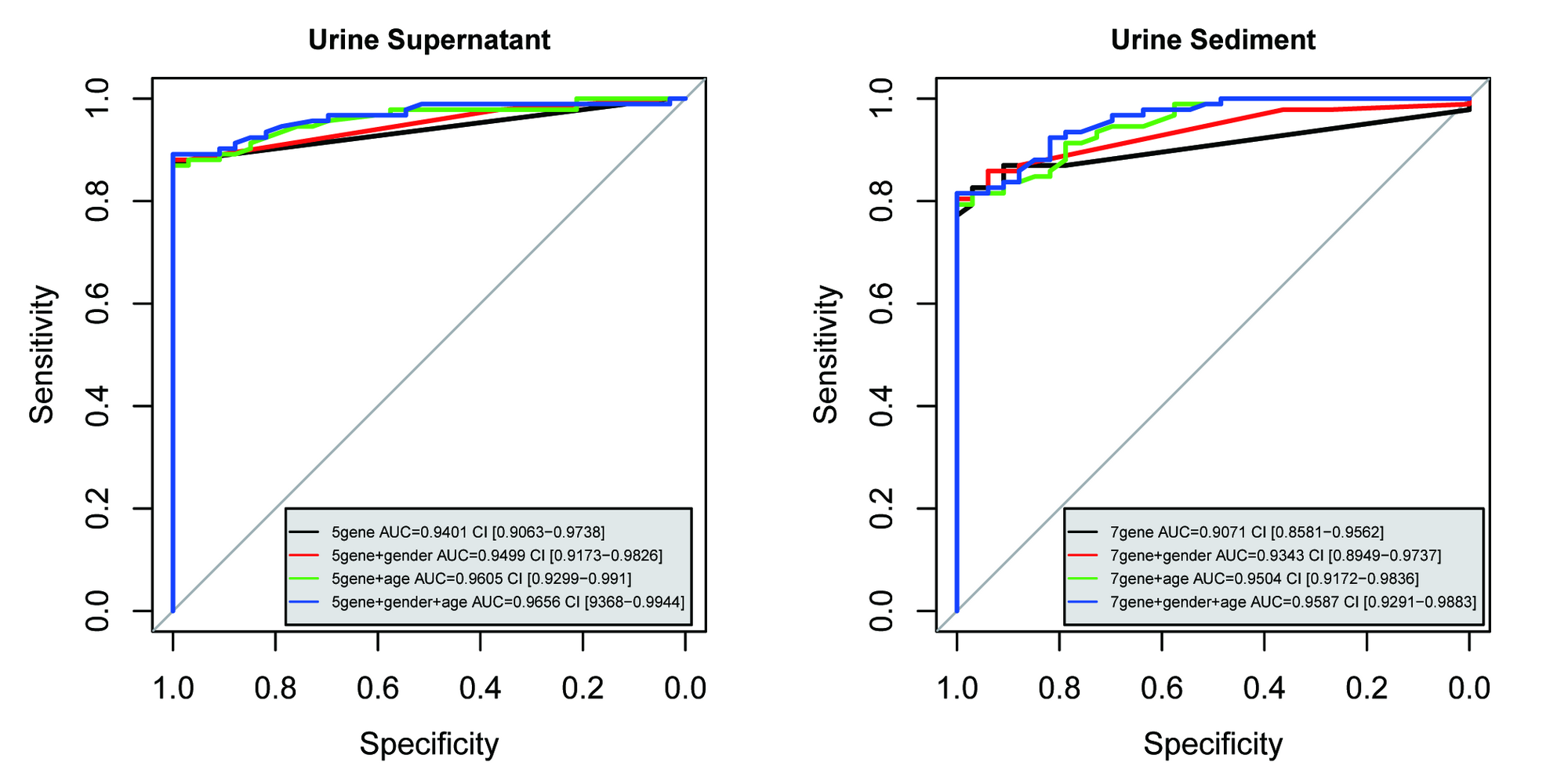

Supplement: Supplementary file 5 — Additional file 5: Figure S4. The AUCs of urine supernatant five-gene and urine sediment seven-gene panels, respectively, in combination with integrated demographic information. With the addition of age and gender, the diagnostic power of the urine supernatant five-gene model slightly improved as the AUC reached 0.9656 (95% CI 0.9368–0.9944) and the diagnostic power of the urine sediment seven-gene model improved as the AUC reached 0.9587 (95% CI 0.9291–0.9883). [file 40169_2020_257_MOESM5_ESM.tif]
